# Exploration and Exploitation in Natural Viewing Behavior

**DOI:** 10.1038/s41598-017-02526-1

**Published:** 2017-05-23

**Authors:** Ricardo Ramos Gameiro, Kai Kaspar, Sabine U. König, Sontje Nordholt, Peter König

**Affiliations:** 1 Institute of Cognitive Science, University of Osnabrück, Osnabrück, Germany; 2 Social and Media Psychology, Department of Psychology, University of Cologne, Köln, Germany; 3 Department of Neurophysiology and Pathophysiology, University Medical Center Hamburg, Eppendorf, Germany

## Abstract

Many eye-tracking studies investigate visual behavior with a focus on image features and the semantic content of a scene. A wealth of results on these aspects is available, and our understanding of the decision process where to look has reached a mature stage. However, the temporal aspect, whether to stay and further scrutinize a region (exploitation) or to move on and explore image regions that were yet not in the focus of attention (exploration) is less well understood. Here, we investigate the trade-off between these two processes across stimuli with varying properties and sizes. In a free viewing task, we examined gaze parameters in humans, involving the central tendency, entropy, saccadic amplitudes, number of fixations and duration of fixations. The results revealed that the central tendency and entropy scaled with stimulus size. The mean saccadic amplitudes showed a linear increase that originated from an interaction between the distribution of saccades and the spatial bias. Further, larger images led to spatially more extensive sampling as indicated by a higher number of fixations at the expense of reduced fixation durations. These results demonstrate a profound shift from exploitation to exploration as an adaptation of main gaze parameters with increasing image size.

## Introduction

Vision is the key modality by which humans interact with the environment. However, our processing capacity is limited regarding attention^1–5^. In fact, visual attention is an integral part of our interaction with the environment. By focusing the line of sight by eye movements, humans actively select regions of interest for in-depth processing with high spatial resolution^6–8^. Therefore, investigating the visual system with an emphasis on overt visual attention has developed into a most active research topic in cognitive science^9^.

Although vision evolves in an alternation of saccades and fixations, overt visual attention is a continuous process. We constantly have to decide whether to move on to sample another image region or to linger in the currently fixated region for in-depth processing. In analogy to other science areas, here we label these two processes *exploration* and *exploitation* respectively^10^. Thus, each decision to fixate on a new location terminates scrutinizing of the currently fixated region and establishes a classic exploration–exploitation dilemma^11, 12^. In visual behavior, the number and spatial distribution of fixations characterize the exploration of a scene^13^. By contrast, the time spent at a fixated location (i.e., fixation duration) reflects the degree of in-depth processing of what is observed and hence characterizes the exploitation aspect^14–16^. However, given time constraints for image observation and interpretation, exploration of the whole visual scene and exploitation of local image regions impose conflicting requirements. Consequently, while scanning a scene, overt attention in visual behavior consists of a continuous interplay between exploration and exploitation.

Vision research has identified several factors that influence eye movement behavior. These factors can be classified as top-down and bottom-up influences^2, 17–20^ as well as spatial viewing biases^21–25^.

Top-down effects are aspects of the observing agent, the task, and the context. In particular, top-down factors comprise the observer’s current motivational state and time-independent personality traits^26, 27^. Furthermore, the observer’s current emotional state^28, 29^ as well as the emotional valence of external objects^30–32^ are strong top-down influences on exploration and exploitation. Top-down factors also cover specific personal interests^33^ that may be different depending on the current task performed by the observer^34, 35^. Overall, such top-down factors play a major role in viewing behavior and can explain a large part of the variance in eye movements.

By contrast, bottom-up factors comprise the properties of the stimulus that influences the selection of fixation locations. These properties may relate to primary contrasts (e.g., luminance, color, and saturation). For instance, edge information and high contrast of image regions play a role in attracting fixations^23, 36–38^. In fact, models based on the concept of a salience map that incorporates such basic image properties can predict human visual behavior with high performance^39^. Furthermore, it has been shown that, even in repetitive stimulus presentations, when the semantic content of the image is already known to a large degree, the low-level impact factors remain influential^23^. Thus, the bottom-up influence of stimulus-dependent features significantly guides visual behavior.

Finally, spatial constraints, partly based on oculomotor properties, lead to spatial viewing biases that also affect eye movement behavior^22^. Motor biases in the saccadic system cause a preference to perform rather short saccades (usually less than 15 degrees of visual angle^40^) while observing scenes^41–43^. This preference for short saccades in combination with a central fixation cross, preceding a trial in typical eye-tracking experiments, is argued to be one reason why researchers commonly observe a tendency to focus on central image regions^44–46^. However, in other image categories, such as webpages, the spatial bias is shifted to upper left areas, due to structural formation of webpages^47^. Consequently, this central tendency significantly determines visual exploration and exploitation.

The separation of these three factors (i.e., top-down, bottom-up, and spatial viewing bias) helps to understand guidance of visual behavior. For the investigation of the relation between exploration and exploitation, these three factors may be varied systematically. However, parametric variations of tasks or stimulus categories are currently not feasible. In contrast, by systematically changing the size of visual scenes, we can examine exploitation and exploration tendencies more directly. Thus, this approach is well suitable for an examination of the exploration–exploitation interplay.

All researchers in the field are confronted with the practical issue of variations of stimulus size. In modern days, media devices are characterized by a wide range of display sizes. Sizes vary from small (e.g., smartphones) to intermediate (e.g., tablets and laptops) to large (e.g. TVs). Hence, the question arises as to what degree we can generalize the results of individual eye-tracking studies based on specific visual display sizes.

A previous study indicated that the image size needs to be considered in eye-tracking research more than has been done previously^48^. Different eye-tracking laboratories commonly use varying settings concerning monitor sizes, image resolution, and viewing distance (for an exemplary selection, see Table 1). These variations in image size may influence viewing behavior. Indeed, von Wartburg *et al*.^48^ found that an increasing image size led to an increase of the mean and median saccade amplitude. Thus, we consider size to be a main property of an image because it determines the size of all depicted objects and their spatial relations. The richness of observable detail attenuates with decreasing size, which might also affect the subjective saliency of basic image features such as contrasts in color and luminance and thus also influence bottom-up properties. Furthermore, depending on the distance to the monitor, the image size directly scales the amount of visual information presented to the observer (see Table 1). Therefore, the influence of different image sizes on exploratory and exploitive viewing behavior has to be considered.

**Table 1:** Exemplary list of stimulus size and resolution of the display screen used in some recent eye-tracking studies.

Study	Stimulus size (width × height) in visual degrees	Area in visual degrees^2^	Fraction of elliptic visual field in percent (200° horizontal and 130° vertical)	Screen distance in cm	Screen resolution (width × height) in pixels
Bindemann^46^	22.0 × 16.0	352	1.72	80	1024 × 768
Henderson *et al*.^35^	24.3 × 18.7	454.41	2.23	90	800 × 600
Einhäuser *et al*.^34^	29.0 × 22.0	638	3.12	80	1024 × 768
Unema *et al*.^16^	31.0 × 26.0	806	3.95	60	1024 × 768
Rauthman *et al*.^27^	33.2 × 25.2	836.64	4.10	58	1280 × 1024
Kienzle *et al*.^49^	35.7 × 27.1	967.47	4.74	60	1024 × 768
Tatler^44^	40.0 × 30.0	1200	5.88	60	1600 × 1200
Kaspar *et al*.^47^	45.7 × 36.6	1672.62	8.19	45	1280 × 1024
von Wartburg *et al*.^48^	10.0 × 7.7	77	0.38	70.5	1600 × 1200
	18.0 × 13.8	248.4	1.21		
	26.0 × 19.8	514.8	2.52		
	34.0 × 26.0	884	4.33		

The stimulus size in visual degrees depends on the distance between participant and screen. Also, the approximate fraction of the (elliptic) visual field area covered by the stimulus is presented.

**Figure 1:**
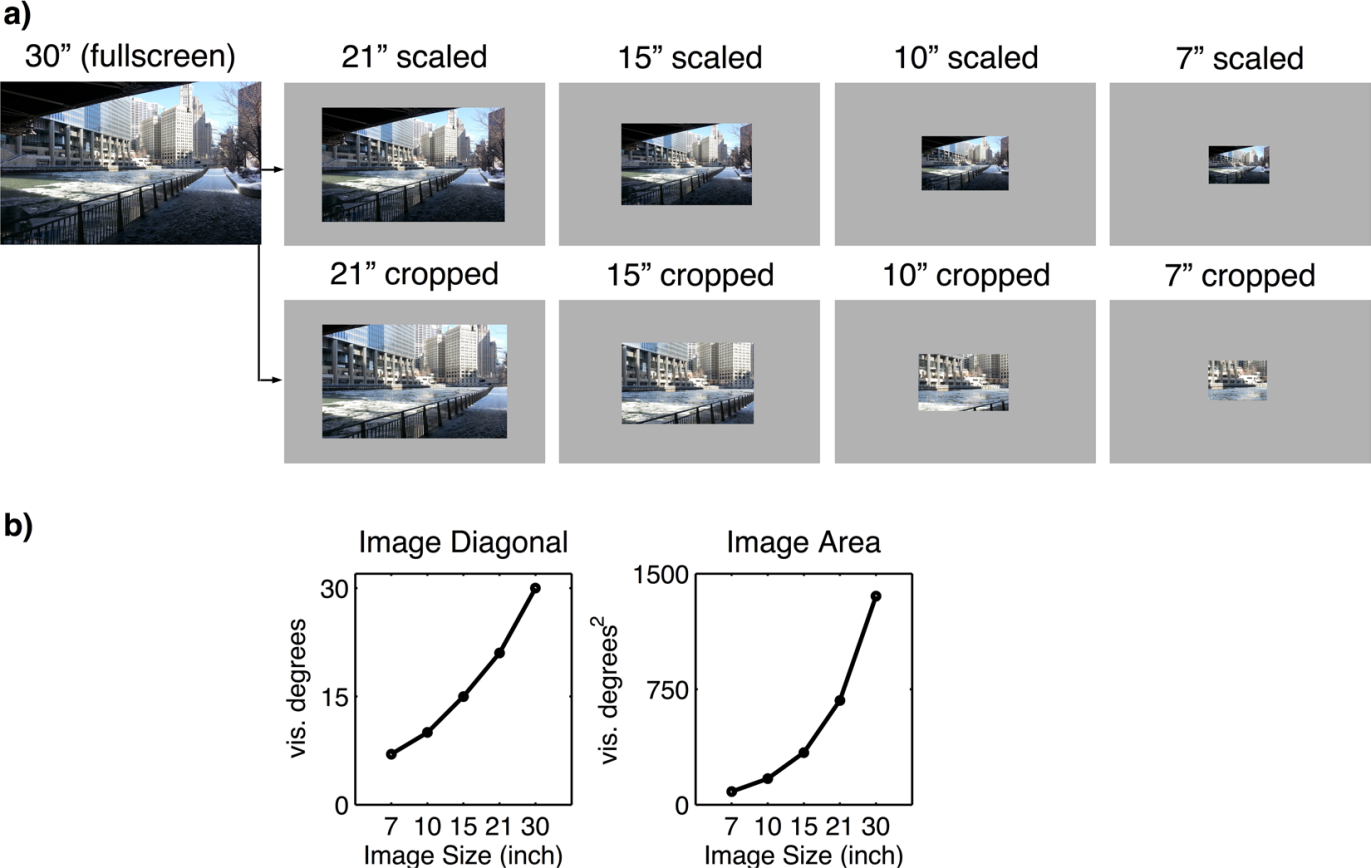
(**a**) Example of an urban image differing in size (scaled: upper row; cropped: lower row). (**b**) Visualization of the increase of image size from 7” to 30” (diagonal and area).

In the present eye-tracking study, we thus investigated the influence of varying image sizes on changes in gaze behavior. In contrast to von Wartburg *et al*.^48^, we used an extended design and a broader set of analyses to examine exploration and exploitation tendencies. We presented urban, natural, and webpage images in five different sizes ranging from 7” to 30” to match the dimensions of typical screen sizes. However, a potential problem with scaling of an image is the change in available visual information. Even when scaled, the power law behavior implies constant statistical properties of the image. Yet, scaling affects sizes of objects and their distances to each other within the image. More specifically, a reduced image size leads to a reduced image resolution and lower spatial details of such objects. To investigate whether changes in gaze behavior are actually a result of the spatial properties of the image with respect to its size or whether changes are instead affected by varying spatial details, we manipulated the images in two complementary ways: in the “scaled condition,” we scaled the images to the targeted screen size, thus scaling the complete visual scene down (Fig. 1a). In the “cropped condition,” we used the complete scene and cropped out the appropriate central section for the smaller image sizes. Hence, in this condition, the visual scene displayed only the central part of the original image in smaller image sizes. We as a result of this guaranteed that the image resolution and depth of details within the scene remained constant. Moreover, we used images of different categories, as image type has been found to influence viewing behavior^23, 26, 50^ strongly. In our study, we used images that showed urban scenes, landscape scenes, and screenshots of webpages. These image categories were chosen according to two reasons: first, conventional eye tracking studies investigating visual behavior on natural scenes usually include urban and landscape images (e.g.ref. 23, 24, 44, 48). Second, webpages form a stimulus class that occurs in many real-world scenarios where people use and interact with technical devices varying in display size^51^. Thus, with the present study design, we were able to examine how exploration and exploitation change in relation to different image sizes and image types.

To assess the exploration of the visual scenes, we examined the spatial bias captured by the central tendency and the entropy of the spatial distribution of fixations. In general, a spatial bias reflects a deviation from a homogenous distribution of fixations across a visual scene. Thereby, the central tendency describes a bias in visual exploration with an increased number of fixations in the central image region at the expense of higher eccentricities. In contrast, the entropy captures the heterogeneity of the distribution of fixations independently of specific geometrical arrangements and hence is a suitable signature of the general degree of exploration of an observer’s viewing behavior^29^. Thus, central tendency and entropy describe different aspects of the spatial distribution of fixations.

Furthermore, we investigated saccade amplitudes and the number of fixations as an additional measure of spatial exploration. For exploitation, we specifically analyzed the distribution of fixation durations, as this measure indicates the time allocated to in-depth processing of fixated regions. Besides an investigation of how fixation durations change across image sizes, we additionally analyzed whether a change in fixation duration would directly result from varying image sizes or, alternatively, from an interdependence between fixation duration and saccade amplitude. Regarding the latter option, divergent results have been reported: Radach and Heller^52^ described fixation durations and saccade amplitudes as independent. In contrast, Unema *et al*.^16^ concluded that fixation durations and saccade amplitudes are controlled by the same mechanism. We thus included an analysis of the interaction between saccadic amplitude and the duration of the preceding fixation. Further, we investigated saccade durations^53, 54^ to determine whether a change in saccade durations across varying image sizes has an impact on the number and duration of fixations, as a consequence of a limited presentation duration of the stimulus. Overall, this selection of eye movement parameters enabled a comprehensive investigation of the trade-off between exploration and exploitation.

Three hypotheses about how image size may affect visual behavior guided our research. First, we hypothesized that an increase of the stimulus size increases the visual exploration by spreading out fixation locations to a larger degree, which should be reflected by a corresponding change in central tendency and entropy. However, assuming constant oculomotor constraints, saccade amplitudes should stay constant, and the exploitation of image regions should not change across image sizes. This yields:

**Hypothesis 1:**   *Visual exploration increases with an increase of the image size, which is reflected by a reduction in the central tendency and an increase of entropy*.

Furthermore, oculomotor constraints might also adapt with varying image sizes. Specifically, an increase of saccadic amplitudes with increasing image size might support increased visual exploration. This yields:

**Hypothesis 2**:   *Visual exploration increases with an increase of the image size, accompanied by an increase of saccade amplitudes*.

As an effect of the adaption of oculomotor constraints to varying image size, and due to the fixed presentation time of the stimuli, we additionally hypothesized that exploitation would decrease with larger image sizes. Instead, on larger images within the same presentation duration more fixations of shorter duration should be performed. This change would exemplify a shift from exploitation to increased spatial exploration of the image. This yields:

**Hypothesis 3:**   *An increase of the stimulus size leads to more fixations of shorter duration per unit time interval, i.e. favoring exploration at the expense of reduced exploitation*.

## Methods

All participants gave written informed consent to participate in this study. We performed the study in accordance with the guidelines of the German Psychological Society. The experimental methods were approved by the Ethical Committee of the University of Osnabrück (Germany).

### Participants

Twenty-four participants (16 female) with a mean age of 21.38 years (*SD* = 3.00) took part in this study. All of them had normal or corrected-to-normal vision and passed the Ishihara test for color blindness^55^. Participants were told to freely observe the different images on the screen.

### Apparatuses

We used a 30” widescreen Apple Cinema HD Display (Apple, California, USA) for stimulus presentation. The screen had a native resolution of 2560 × 1600 pixels. Participants were seated in a darkened room at a distance of 80 cm from the monitor. The seating distance resulted in 55 pixels per visual degree. We did not fixate the subject’s head with a headrest in order to facilitate comfortable conditions. However, the experimenter verbally instructed the subjects not to make head movements during the experiment.

We recorded eye movements using a head-mounted Eye Link II eye-tracker (SR Research Ltd.). The eye tracking system enclosed three cameras: Two eye cameras (one for each eye) and one head camera. The head camera recorded fixed infrared sensors attached to the corners of the monitor, to constantly calculate the head position in relation to the screen. This guaranteed stable gaze recordings based on eye movements, independent of residual involuntary head movements. For validation, we found in a separate paradigm that head movements of up to 10° lead to an eye drift of less than 0.5°. This matches approximately the accuracy of the eye tracker with respect to the averaged drift based on the calibration. Thus, the eye tracker well compensates for the investigated range of head movements.

In order to calibrate the system, each participant had to fixate on 13 black circles that appeared consecutively at different screen locations. The size of the point was about 0.5°. The calibration was validated afterwards by calculating the drift error for each point. The calibration was repeated until the system reached an average accuracy of <0.3° for at least one eye. The eye with lower validation error was automatically detected by the system and tracked. We conducted monocular recordings with a sample rate of 500 Hz. Fixation locations and times were calculated online by the eye-tracker. Saccade detection was based on a velocity of at least 30°/s and an acceleration of at least 8000°/s^2^. Either one (or both) of these criteria had to be met, to trigger a saccade signal. This saccade signal had to be sustained for at least 4 ms for a saccade to be detected. The temporal and spatial onset of the saccade was defined when the eyes significantly moved from the fixation point, i.e. exceeding a motion threshold. By default, we set this motion threshold to 0.1°. After saccade onset, the minimal saccade velocity was 25°/s. Fixations were defined as periods without saccades.

### Stimuli

We used 360 static images assigned to three different categories. The first category covered landscape images depicting natural environments like open landscapes, forests, or flowers, with an absence of any human-made objects. The second category covered urban images showing, for example, house exteriors, streets, and vehicles. The stimuli of these two categories were used in several previous studies^23, 26, 29^, and images of these kinds are widely used in eye-tracking research^56–58^. The third category contained screen shots from existing webpages provided by the EyeQuant company (WhiteMatter Labs GmbH). To avoid habitual viewing patterns, we only used webpages that were not highly popular like major news feeds. Webpages were included in the study as they are also stimuli of high interest in the context of eye movement analyses^33, 59, 60^. They were also included due to their different geometrical structure, which leads, among other things, to the relocation of the central fixation bias found for natural images^44–46^ to the left upper corner among European participants^47, 50^. All images were scaled up to 2560 × 1600 pixels by bicubic interpolation. The 30” images were resized to smaller image sizes during the experiment. The stimulus size reduced gradually according to the following equations:

1 $${Y}_{d}=\frac{1}{{(\sqrt{2})}^{L}}\ast 1600$$

2 $${X}_{d}=\frac{1}{{(\sqrt{2})}^{L}}\ast 2560$$

where *X*_*d*_ and_*d*_ are the two-dimensional coordinates of the desired stimulus resolution, and *L* denotes the level of size reduction that had to be calculated based on the full-size screen resolution (30” diagonal; 46.5° × 29.1°). In addition to the 30” images, we computed four more levels of stimulus size, which resulted in scaled sizes of 1810 × 1131 pixels (~21” diagonal; 32.9° × 20.6°), 1280 × 800 pixels (~15” diagonal; 23.3° × 14.5°), 905 × 565 pixels (~10” diagonal; 16.5° × 10.3°), and 640 × 400 pixels (~7” diagonal; 11.6° × 7.3°) (Fig. 1a). The background color for images that were smaller than full size was set to neutral gray (RGB: 128/128/128). In contrast to the scaled images, the cropped images were realized by extracting a corresponding central section of the original 30” images (Fig. 1a). Figure 1b visualizes the increase of image size.

### Procedure

**Figure 2:**
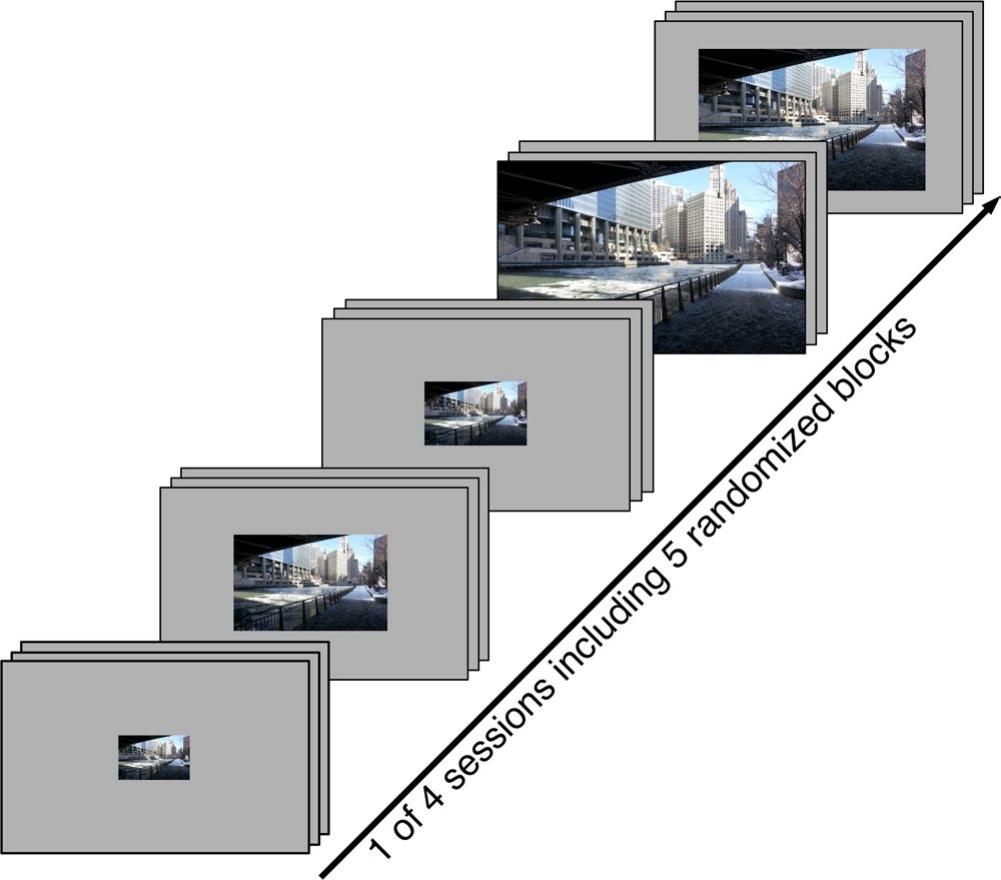
Structure of one of four experimental sessions with randomized order of blocks. Within each block, 18 images (6 images per category) of the same size were presented for 6 s each.

The experiment was divided into four equally long sessions (90 images per session), each consisting of five blocks (Fig. 2). Per block, we presented 18 equally sized images (six of each image category) in a random order, where half of them were cropped and half of them were scaled. Each image was randomly assigned to one of the five possible image sizes and to the scaled or cropped condition. For statistical analyses, half of the 30” images observed by one participant were randomly assigned to the scaled condition, while half of them were assigned to the cropped condition. The blocks were also presented in random order within a session and across participants. Each image of the whole stimulus set occurred only once per participant. The images were centered on the screen and presented for 6 s each, following previous studies^23, 26, 29^. Between trials, participants had to fixate a central dot used for drift correction and to reset the eye position to the center of the screen.

### Dependent variables

We computed several eye-tracking parameters, including the number of fixations, the mean duration of single fixations, saccade amplitude, and the distribution of fixations regarding central tendency and entropy.

#### Single fixations and saccade amplitude

For the number of single fixations, we added up all fixations within a trial. However, the first fixation was excluded from the analysis as it was a direct consequence of the preceding fixation dot used for drift correction. We also excluded invalid fixations that were placed outside the image borders of the respective image size (7”: 2.65%; 10”: 2.20%; 15”: 1.59%; 21”: 1.15%; 30”: 1.08%).

The duration of a fixation was calculated by subtracting its temporal onset from its temporal offset. Fixations with a duration of less than 50 ms (2.16% of all fixations) or more than two standard deviations above the grand mean over all participants (cut-off at 649.72 ms; 3.27% of all fixations) were excluded to avoid biased results that could derive from outliers^23, 26^. All remaining fixations that did not meet our exclusion criteria were labeled as valid fixations.

Saccade length was operationalized by the Euclidean distance between two consecutive fixations marked by their coordinates in the two-dimensional image space. We excluded invalid saccades when either the pre-saccadic or post-saccadic fixation was located outside of the image region (7”: 4.40%; 10”: 3.64%; 15”: 2.61%; 21”: 1.86%; 30”: 1.69%).

#### Fixation density maps and entropy

**Figure 3:**
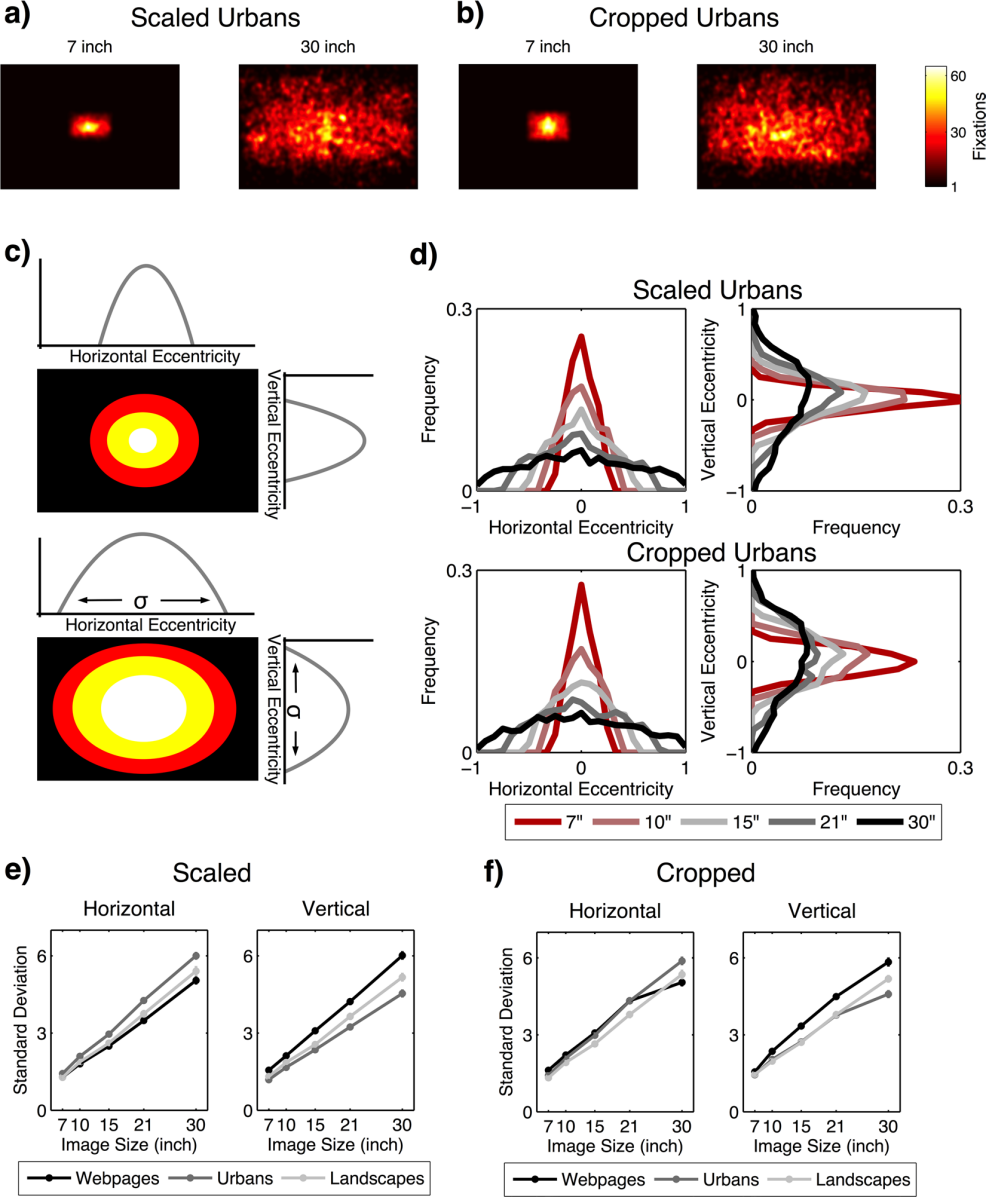
The distribution of fixations in terms of a fixation density map in the two-dimensional image space for (**a**) urban image in the scaled condition and (**b**) cropped condition. (**c**) Sketch showing the eccentricities of the fixation distribution in horizontal and vertical directions. The extent of the eccentricity was measured by the standard deviation. (**d**) Eccentricities of urban images in the scaled and cropped condition in horizontal and vertical directions for all image sizes. Plots for scaled (**e**) and cropped (**f**) images indicate the extent of the eccentricities measured by the standard deviation depending on image category and image size. Error bars indicate the standard error of the mean.

We characterize the spatial bias in the form of the central tendency separately for both cardinal directions. That is, we computed the marginal distribution of fixations in the horizontal and vertical directions within the two-dimensional image space (Fig. 3c). By calculating the standard deviation of these distributions, we estimated the magnitude of eccentricity along the vertical and horizontal axes, respectively. This procedure amounts to the root-mean-square distance of fixations from the center of gravity of all fixations. When the standard deviation in horizontal and vertical direction is small, fixations are concentrated near the center, indicating a strong central tendency. When the standard deviations are large, the fixations are more evenly distributed in the visual field and the central tendency is weak.

To investigate the fixation distribution independently of specific geometrical arrangements, we employed the concept of entropy. We convolved the spatial distribution of fixations (fixation density map, FDM) with a Gaussian kernel. The full width at half maximum (FWHM) of the Gaussian kernel defining the size of the patch was set to 1° of visual angle^26, 29–31, 47^. We selected the size of the Gaussian kernel to approximately match the size of the central part of the fovea. Therefore, and because absolute entropy values depend on the kernel’s size^29^, it was set constant for all image sizes. The entropy *E* of the resulting fixation density map *x* for participant *p* observing image *i* was calculated by means of the standard MATLAB function (MathWorks, Inc.) according to the following equation^26, 29, 47^.

3 $$E(i,p)=-\,\sum FDM(x,i,p)\,\ast \,lo{g}_{2}FDM(x,i,p)$$

A higher entropy value indicates a more spread out fixation distribution and hence a more exploratory viewing behavior. In the next step, we applied a straightforward bootstrapping technique to the entropy values, since estimators of entropy are generally influenced by sample sizes^61^ and, in the domain of eye-tracking data, by a smaller number of fixations^62^. We randomly sampled nine fixations out of all valid fixations made in a trial and calculated the corresponding entropy value. This process was repeated 500 times, and we finally calculated the mean entropy *E* of participant *p* on image *i* according to previous studies^26, 30, 31, 47^. The target sample size of nine fixations was selected according to previous studies that also analyzed entropy on natural images presented for 6s^26^ in order to maintain sufficient statistical power^62^ and to keep the number of excluded trials with fewer fixations to a minimum (5.08%).

Please note, that the central tendency and entropy characterize two distinct measures that can yield independent results. For example, a subject may fixate two regions of the image outside of the central part with large spatial distance. However, within each image region, the subject makes several fixations that are close together. Thus, the central tendency will be weak, as the average distance of the fixated image regions from the center is large. However, the entropy will be small indicating a clustering of fixations in only a small fraction of image regions.

## Results

### The spatial bias as captured by the central tendency and entropy

Our first hypothesis stated that visual exploration increases with increasing image size, which is reflected by a reduction of the central tendency and an increase of entropy. Therefore, we investigated the distribution of fixations for each image size. The resulting two-dimensional density maps of fixations (Fig. 3a and b) capture the corresponding data.

First, we analyzed the central tendency assessed by the eccentricities of the fixation distribution in horizontal and vertical directions. The extent of the eccentricity was measured by the standard deviation (see Methods). Based on these values, we calculated two 2 (image condition: scaled verses cropped) × 3 (image category: webpages versus urban images versus landscape images) × 5 (image size) repeated measures ANOVA (Greenhouse-Geisser applied) for the horizontal and vertical eccentricity, respectively. With respect to the horizontal direction, we found a main effect of image size reflecting a reduction of the central tendency (i.e., increasing standard deviations) with increasing image size. This is compatible with the first hypothesis. However, as shown in Table 2, we also found significant two-way and three-way interactions. Hence, we computed a set of contrasts comparing all pairs of image sizes (Bonferroni-adjusted alpha level: *α* = 0.0008) separately for each image category and the two image conditions. The results showed that the central tendency in horizontal direction continuously decreased with increasing image size for all image categories and both image conditions [all *t*s ≥ 6.671; *p*s < 0.0008] (Fig. 3e and f). Hence, the significant interactions derived from different effect sizes; for example, the decrease of the central tendency from 21” to 30” images in horizontal direction was less pronounced on cropped webpages compared to other contrasts. For a detailed presentation of all paired contrast, see Table S1 in the online supplementary file.

**Table 2:** Results of the 2 × 3 × 5 (image condition × image category × image size) ANOVA for the central tendency in the *horizontal* direction.

Effect	*F*	*p*	*η*_*p*_^2^
**Main effects**			
Image condition	44.207	<0.001	0.658
Image category	52.366	<0.001	0.695
Image size	762.114	<0.001	0.971
**Two-way interactions**			
Image condition × image category	46.106	<0.001	0.667
Image condition × image size	15.081	<0.001	0.396
Image category × image size	40.557	<0.001	0.638
**Three-way interaction**			
Image condition × image category × image size	7.144	<0.001	0.237

With respect to the eccentricity in vertical direction, the ANOVA revealed a main effect of the image size, which was again qualified by image category and image condition, as indicated by significant two-way interactions (see Table 3). However, paired *t*-tests (see Table S2 in the online supplementary file) again showed that the strength of the central tendency in vertical direction continuously decreased with increasing image size in all image categories and both image conditions [all *t*s ≥ 8.953; *p*s < 0.0008].

**Table 3:** Results of the 2 × 3 × 5 (image condition × image category × image size) ANOVA for the central tendency in the *vertical* direction.

Effect	*F*	*p*	*η*_*p*_^2^
**Main effects**			
Image condition	26.669	<0.001	0.537
Image category	201.006	<0.001	0.897
Image size	730.583	<0.001	0.969
**Two-way interactions**			
Image condition × image category	4.493	<0.050	0.163
Image condition × image size	9.251	<0.005	0.287
Image category × image size	74.004	<0.001	0.763
**Three-way interaction**			
Image condition × image category × image size	1.700	0.174	0.069

Overall, we found that the central tendency decreased with image size for all image categories and conditions, supporting Hypothesis 1.

To investigate whether this change in central tendency was linear, we applied a lack-of-fit *F*-test comparing the curve of the measured data, the standard deviation of the spatial distribution, with a linear regression fitted to these data. Thus, we investigated whether the linear model was an adequate estimate for the observed data. In the scaled conditions, the results showed no deviation from linearity in either the horizontal or the vertical direction across all image categories [all *F*s ≤ 1.193, *p*s ≥ 0.316, *R* ^2^ ≥ 0.918]. This indicates that the linear model estimated the observed data adequately. In the cropped condition, the results revealed no deviation from linearity in either direction for landscape images [both *F*s ≤ 0.501, *p*s ≥ 0.682, *R* ^2^ ≥ 0.925]. In the urban category, results showed no deviation from linearity in the horizontal direction only [*F* = 1.740, *p* = 0.163, *R* ^2^ = 0.955], whereas in the vertical direction, the observed data deviated significantly from linearity [*F* = 9.159, *p* < 0.001, *R* ^2^ = 0.916]. In fact, as shown in Fig. 3f, the curve for urban pictures in the vertical direction showed a slight flattening between 21” and 30” images, and thus it is not fully described by a linear decrease of the central tendency. However, in general, the slope followed a linear trend, as also indicated by the high *R* ^2^ of 0.916. With respect to cropped webpages, the deviation between the observed data and the linear regression model was significant in both directions [both *F*s ≥ 6.316, *p*s ≤ 0.001, *R* ^2^ ≤ 0.942]. Again, Fig. 3f shows that the curves representing an increase of the spatial bias on webpages in both directions showed a slight flattening between 21” and 30” images, but still represent a rather linear trend [both *R* ^2^ ≥ 0.919]. Overall, in both the scaled and cropped condition, the standard deviation of the spatial distribution of fixations increased in a mainly linear fashion fully compatible with the Hypothesis 1.

**Figure 4:**
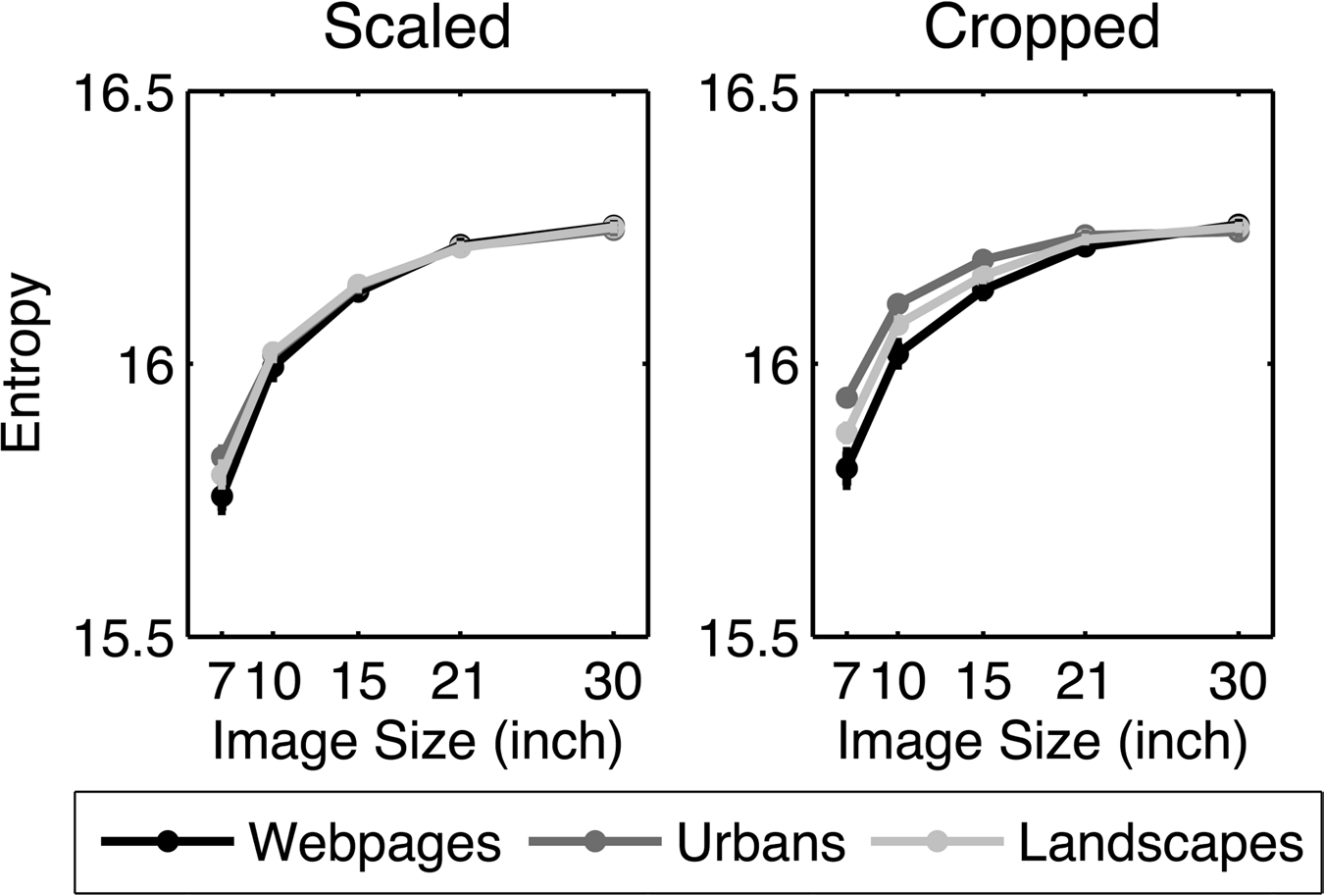
Geometrically independent distribution of fixations in image space operationalized by entropy for each image category and size. Error bars indicate the standard error of the mean.

In the next step, we analyzed the entropy quantifying the extent of exploratory viewing behavior independently of specific geometrical arrangements. We calculated the entropy for each participant in each trial in order to run the 2 × 3 × 5 (image condition × image category × image size) repeated measures ANOVA. We found a main effect of the image size, compatible with Hypothesis 1. This main effect was further qualified by image category and image condition (Fig. 4), as shown by two-way and three-way interactions (see Table 4). Paired *t*-tests (Bonferroni-adjusted alpha level: *α* = 0.0008) showed that the entropy continuously increased with increasing image size in all image categories and both image conditions [all *t*s ≥ 7.688; *p*s < 0.0008], except the step from 21” to 30” urban images in the cropped condition. For a detailed presentation of all paired contrast, see Table S3 in the online supplementary file.

**Table 4:** Results of the 2 × 3 × 5 (image condition × image category × image size) ANOVA for entropy.

Effect	*F*	*p*	*η*_*p*_^2^
**Main effects**			
Image condition	165.714	<0.001	0.878
Image category	9.009	<0.005	0.281
Image size	799.499	<0.001	0.972
**Two-way interactions**			
Image condition × image category	22.905	<0.001	0.499
Image condition × image size	55.787	<0.001	0.708
Image category × image size	9.254	<0.001	0.287
**Three-way interaction**			
Image condition × image category × image size	3.492	<0.050	0.132

We again compared the observed data with a linear regression model by means of a lack-of-fit *F*-test. In the scaled condition, the results showed a significant deviation from linearity for all image categories [all *F*s ≥ 43.923, *p*s ≤ 0.001, *R* ^2^ ≤ 0.733]. Indeed, Fig. 4 suggests that the increase of entropy across image sizes followed a rather logarithmic trend. Therefore, we used a lack-of-fit *F*-test comparing the observed data with a log-linear regression model. For this, we computed the natural logarithm for each image size. We then applied a linear regression to the log-transformed data and tested whether the linear model was adequate to describe the log-transformed data. The results of the lack-of-fit *F*-test again revealed a significant deviation from linearity in the log-transformed data for all image categories [all *F*s ≥ 16.377, *p*s ≤ 0.001, *R* ^2^ ≤ 0.864]. Hence, the entropy did not increase logarithmically with increasing image size in the scaled condition. Yet, correlations for all image categories indicated that the log-linear regression model was a slightly better model for describing the observed data [all *R* ^2^ ≥ 0.763] than the linear model [all *R* ^2^ ≤ 0.733].

In the cropped condition, the results of the linear regression model revealed a significant deviation from linearity for all image categories [all *F*s ≥ 27.128, *p*s ≤ 0.001, *R* ^2^ ≤ 0.689]. Again, Fig. 4 indicates that the slope was logarithmic rather than linear. Therefore, we tested the linear regression model with the log-transformed data. The results revealed a significant deviation from linearity in the log-transformed data [all *F*s ≥ 9.631, *p*s ≤ 0.001, *R* ^2^ ≤ 0.827]. Hence, the entropy did not increase logarithmically with increasing image size in the cropped condition. However, correlations for all image categories indicated that the log-linear model was more adequate for describing the data [urban images & landscapes: both *R* ^2^ ≥ 0.826; webpages: *R* ^2^ = 0.712] than the linear model [urban images & landscapes: both *R* ^2^ ≤ 0.680; webpages: *R* ^2^ = 0.607]. Overall, the entropy generally increased with increasing image size in both the scaled and cropped conditions, but not in an exclusively logarithmic fashion.

To summarize, visual exploration in terms of the distribution of fixations showed a decrease with respect to the central tendency and an increase of entropy with increasing image size best but not completely described by a logarithmic function. These signatures were comparable between the scaled and the cropped conditions and occurred across all image categories, providing strong support for Hypothesis 1.

### Saccade amplitude

**Figure 5:**
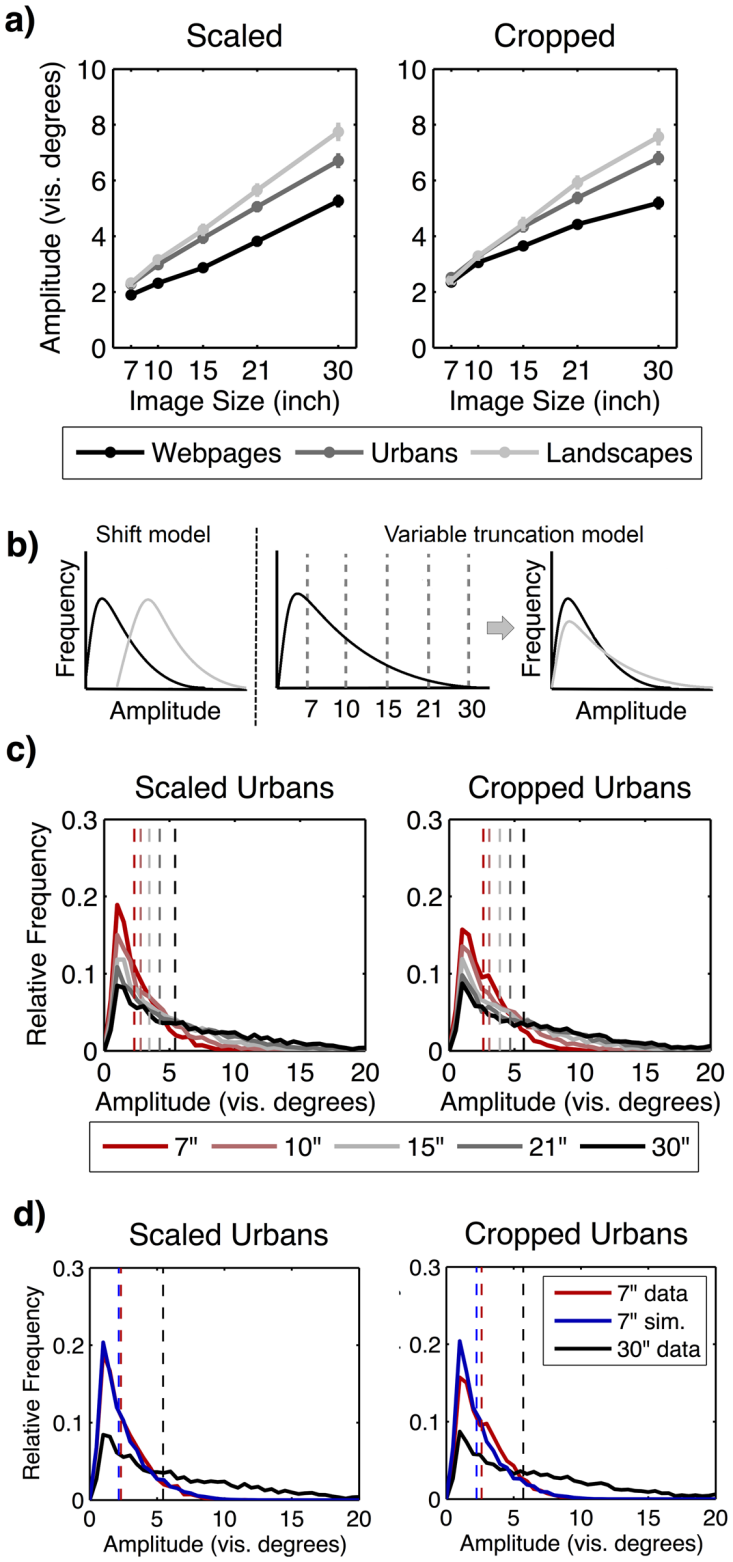
(**a**) Increase of the mean saccade amplitude across image sizes depending on image category and condition (scaled vs. cropped). Error bars indicate standard error of the mean. (**b**) Sketch of the two alternative models describing how the distribution of saccade amplitudes would change with varying image size. (**c**) Real distribution of saccade amplitudes on urban images for each image size in scaled and cropped conditions. Vertical dashed lines indicate the median saccade amplitude. (**d**) Real distribution of saccade amplitudes on 7” urban images and 30” urban images and the simulated distribution for 7” urban images based on saccades sampled from 30” urban images.

The next set of analyses addressed changes in the distribution of saccadic amplitudes. As we found that visual exploration in terms of central tendency and entropy scaled with image size, we evaluated whether saccade amplitude contributed to these changes in exploration. For the analysis of the mean saccade amplitude, we again computed a 2 × 3 × 5 (image condition × image category × image size) ANOVA. We found three main effects but also significant two-way and three-way interactions (see Table 5). Subsequent contrasts comparing all pairs of image sizes (Bonferroni-adjusted alpha level: *α* = 0.0008) separately for each image category and the two image conditions showed that saccade amplitudes continuously increased with increasing image size independent of image category and image condition [all *t*s ≥ 6.629; *p*s < 0.0008, see Table S4 in the online supplementary file]. Hence, the significant interactions reflected different effect sizes associated with changes in image size (see Fig. 5a). Overall, we found that the saccade amplitudes continuously increased with image size for all image categories and conditions, supporting Hypothesis 2.

**Table 5:** Results of the 2 × 3 × 5 (image condition × image category × image size) ANOVA for saccade amplitudes.

Effect	*F*	*p*	*η*_*p*_^2^
**Main effects**			
Image condition	198.224	<0.001	0.896
Image category	119.327	<0.001	0.838
Image size	1075.426	<0.001	﻿0.979
**Two-way interactions**			
Image condition × image category	20.031	<0.001	0.465
Image condition × image size	29.219	<0.001	0.560
Image category × image size	65.063	<0.001	0.739
**Three-way interaction**			
Image condition × image category × image size	2.795	<0.050	0.108

In order to classify the slope of the increasing saccade amplitude, we again used a lack-of-fit *F*-test that compared the measured data with a linear regression model. The results revealed no deviation from linearity in any of the image categories in the scaled condition [all *F*s ≤ 0.992, *p*s ≥ 0.399, *R* ^2^ ≥ 0.846], indicating that the linear regression model was an adequate model for describing the slope of the observed data. In the cropped condition, the results revealed a significant deviation from linearity for webpages only [*F* = 3.941, *p* = 0.010, *R* ^2^ = 0.780], but not for urban and landscape images [both *F*s ≤ 1.821, *p*s ≥ 0.147; *R* ^2^ ≥ 0.849]. Indeed, Fig. 5a shows that, in the cropped condition, the increase of the mean saccade amplitude for webpages did not coincide with the progression of the curves in the urban and landscape categories, but rather flattened with increasing image size. Therefore, we tested whether the curve for webpages followed a logarithmic trend. We computed the natural logarithm for each image size and computed a linear regression for the log-transformed data. The lack-of-fit *F*-test revealed no significant deviation from linearity in the transformed data [*F* = 0.877, *p* = 0.455, *R* ^2^ = 0.796], thus indicating a logarithmic trend in the original data. Overall, our results indicate a linear increase of the mean saccade amplitude with increasing stimulus size for all image categories in the scaled condition, as well as for urban and landscape images in the cropped condition.

The increase of the mean saccade amplitude for larger images could have different sources. On the one hand, with increasing image size, the distribution of saccade amplitudes could be shifted toward longer saccades (*shift model*, Fig. 5b). That is, the probability of a specific saccadic amplitude p(a_sac_, s_image_) while viewing an image of a specific size (s_image_) is determined by an additive constant: p(a_sac_, s_image_) = p(a_sac_ + κ(s_image_), 7”). Here κ is only a (linear) function of image size but independent of the saccadic amplitude. This naive model adds a constant κ to all saccadic amplitudes. On the other hand, observed distributions of saccade amplitudes when viewing images of different sizes could be derived from a single underlying distribution. We defined the corresponding *variable truncation model* (see Fig. 5b) by the following properties: the oculomotor system produces saccadic amplitude candidates characterized by a single distribution, independent of image size. However, saccade candidates to targets outside the image region are always discarded. Furthermore, saccade candidates targeting regions within the viewed image are discarded with a probability proportional to 1 minus the spatial bias of that region (Fig. 5b). This model, by construction, produces a distribution of saccadic targets compatible with the spatial bias observed on smaller images. Whether the generated distribution of saccadic amplitudes matches the observed distribution is an empirical question addressed below.

In a first step, we calculated the distribution of observed saccades for all image sizes. For this analysis, we binned the saccade amplitudes into steps of 0.5°, with the visual angle ranging from 0° up to 20°. We then normalized all frequencies of the saccade amplitudes by dividing the absolute frequencies of each bin by the total number of saccade amplitudes, resulting in probability distributions. This procedure was done individually for each subject and separately for each image category in both conditions. We then averaged the probability distributions over subjects individually for each image size and image condition obtaining the mean distribution of saccade amplitudes (Fig. 5c). Further, we calculated the variance of each probability distribution, which was required for statistical analyses. For the comparison of two distributions (*dist1* & *dist2*), we used chi-squared tests including the difference of means (Δ*μ*) and the overall variance (Δσ^2^) of the tested distributions:

4 $$\,{\rm{\Delta }}{\rm{\sigma }}=\sqrt{\frac{{\sigma }_{dist1}^{2}}{n}\,+\,\frac{{\sigma }_{dist2}^{2}}{n}}$$

with

5 $${\rm{\Delta }}\mu ={y}_{dist1}-{y}_{dist2}$$

and

6 $${\chi }^{2}=\sum _{i=1}^{bins}(\frac{{\rm{\Delta }}{\mu }_{i}^{\,2}}{\Delta {\sigma }_{i}^{2}})$$

Please note that larger *χ* ^*2*^ values indicate a higher difference between the two compared distributions. This test weights the squared difference of means at a specific saccadic amplitude by the inverse of the variance, thus giving higher weight to those amplitudes with higher certainty.

In order to check whether the data were compatible with the shift model, we compared the observed distribution of saccades on 7” images with the distribution observed on 30” images. As shown by Fig. 5c, a significant difference between the two distributions existed for all image categories in the scaled condition [all *χ* ^2^ > 1544.064; *p*s < 0.001] and in the cropped condition [all *χ* ^2^ > 1157.589; *p*s < 0.001]. Hence, the data did not support the shift model.

**Figure 6:**
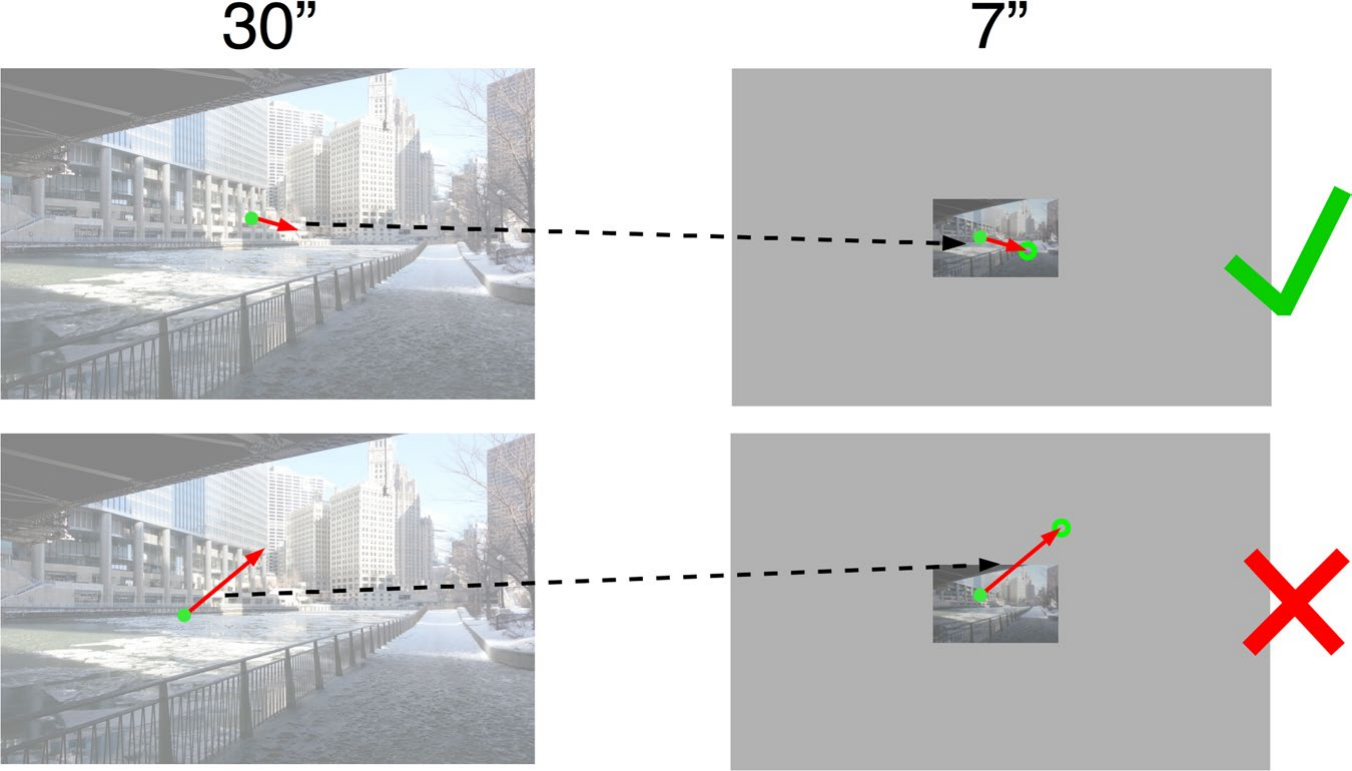
Graphical illustration of the sampling strategy applied to create the simulated distribution of saccade amplitudes of 7” images. Amplitude and angular direction of a randomly chosen saccade made on a 30” image was applied on a randomly selected fixation made on a 7” image. When the target of the corresponding saccade fell into the image coordinates of the 7” image, the saccade was “accepted” for the simulated distribution of saccade amplitudes (upper row). When the target position of the saccade was outside the image coordinates of the 7” image, the saccade was “rejected” (lower row).

For a quantitative description of the variable truncation model, we pooled the saccades for the 30” images for each image category. This distribution served as an approximation of the basic probability distribution of saccadic amplitude candidates independent of image size. Then, we randomly sampled saccades from this pool and applied each sampled saccade to a randomly sampled fixation of a 7” image of the same image category from which we sampled the saccade. The sampling process was done using a bootstrapping method producing 10,000 candidate saccades. From these candidates, we rejected sampled saccades when their pointing target, based on the given fixation, exceeded the border of the 7” image (Fig. 6). Further, we integrated information of the spatial bias of the desired image size. Saccade candidates were more likely not to be rejected proportional to the values of the spatial bias in the target region: we used the fixation distribution in horizontal and vertical direction characterized by the spatial bias (Fig. 3d) as a probability model, showing how likely it was that a fixation would occur at the respective spatial locations across the image dimensions (probability ranged from 0 to 1). We then sampled a random number in the interval between 0 and 1. Fixations were only accepted if the random number was lower than the calculated likelihood of a fixation occurring at the target location based on the spatial bias. With the accepted saccades, we again computed a probability distribution. Similar to the observed data distributions, we calculated these simulation models individually for each subject with respect to his or her observed data. We then averaged over subjects (individually for each image size and image condition) and calculated the mean simulated distributions, as well as the variances of these mean distributions. These computational steps defined the variable truncation model.

In the scaled condition, comparing the variable truncation model with the real distribution of saccades observed on 7” images revealed a good match with no significant difference for urban images [*χ* ^2^ = 43.406, *p* = 0.289] (Fig. 5d). For webpages and landscape images a reasonable fit was achieved as well, yet a significant difference remained [both *χ* ^2^ ≥ 77.229, *p*s < 0.001]. As a control, the comparison between the simulated distribution of saccades for 7” images and the real distribution of saccades on 30” images was huge and significantly different for all image categories [all *χ* ^2^ > 1674.026, *p*s < 0.001]. Thus, the simulated distribution of saccade amplitudes in the variable truncation model (7” sim. in Fig. 5d) significantly deviated from the real distribution of saccade amplitudes observed on 7” webpages and landscape images as well as from the real distribution of saccades observed on 30” images from each image category. However, importantly, the chi-square values representing the model fit showed that our simulated distribution described the actually observed saccades on 7” images much more accurately for all image categories [all *χ* ^2^ ≤ 227.469] than did the distribution of saccades found on 30” images [all *χ* ^2^ ≥ 1674.026], as exemplarily shown for urban images in Fig. 5d. Overall, the simulated distribution served well to explain the saccade amplitudes observed on 7” urban images. With respect to webpages and landscape images, the simulation also provided a more reliable model for explaining the distribution of saccade amplitudes observed on 7” images than did the distribution of saccade amplitudes found on 30” images.

In the cropped condition, we found a significant difference for all image categories when comparing the real distribution with the simulated distribution for 7” images [all *χ* ^2^ ≥ 78.945, *p*s* < *0.001]. Comparing the simulated distribution with the real distribution observed on 30” images also led to a huge and significant difference for all image categories [all *χ* ^2^ ≥ 1422.864, *p*s* < *0.001]. However, once again, the simulation was still a much more reliable model for predicting the real saccade distribution observed on 7” images [all *χ* ^2^(39) ≤ 104.801] compared to the real distribution observed on 30” images [all *χ* ^2^(39) ≥ 1422.864], as indicated by smaller chi-square values (cf. Fig. 5d).

Overall, in both the scaled and cropped condition, we could explain the real distribution of saccade amplitudes observed on 7” images by extracting small saccade amplitudes from the distribution observed on 30” images. This suggests that the distribution of saccadic amplitudes can be explained by subsampling saccades observed for larger stimuli. Apparently, the data suggest constant oculomotor constraints, instead of a true adaptation of oculomotor behavior to varying images sizes (Hypothesis 2).

### Number of fixation and fixation duration

Hypothesis 3 states that an increase in the stimulus size leads to a shift from exploitation with fewer fixations of longer duration to exploration with more fixations of shorter duration.

**Figure 7:**
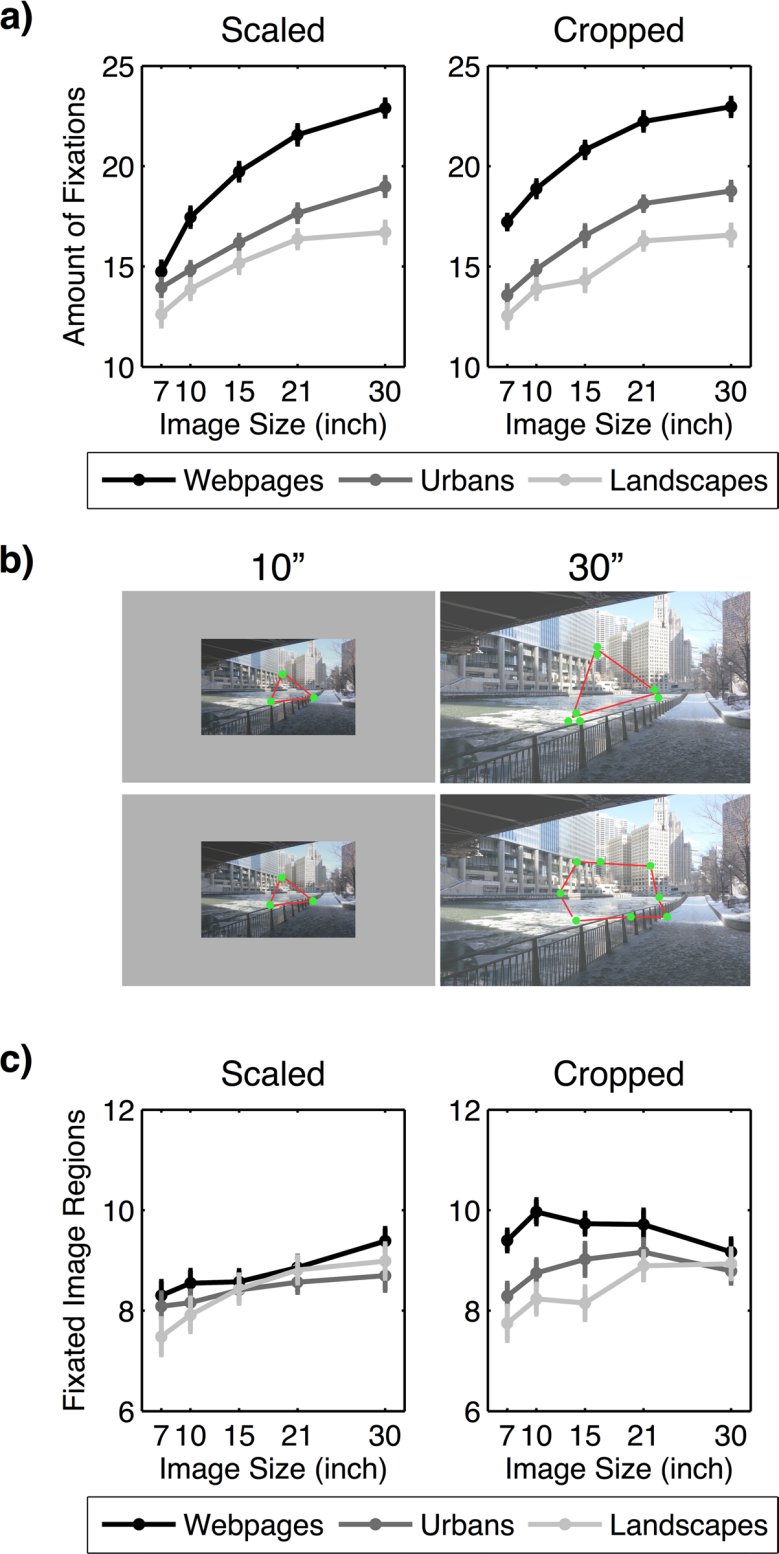
(**a**) Increase in the number of fixations across image sizes depending on image category for the scaled and cropped conditions. Error bars indicate the standard error of the mean. (**b**) Graphical illustration showing that a higher number of fixations on large images might have led to a re-fixation of already seen image regions (upper row) or to a spatially more extensive exploration of the image (lower row). (**c**) Mean number of fixated image regions depending on image size and image category for the scaled and cropped conditions. Error bars indicate the standard error of the mean.

We investigated visual exploration by comparing the total number of fixations across image categories and sizes. We again computed a 2 × 3 × 5 (image condition × image category × image size) ANOVA revealing significant main effects and interactions (see Table 6 and Fig. 7a). The set of contrasts comparing all pairs of image sizes (Bonferroni-adjusted alpha level: *α* = 0.0008) separately for each image category and the two image conditions showed that the number of fixations increased with increasing image size on scaled webpages [all *t*s ≥ 4.979; *p*s < 0.0008]. On cropped webpages, as well as landscape and urban images in both image conditions, results also revealed a continuous increase of the number of fixations with increasing image size [all *t*s ≥ 3.874; *p*s < 0.0008], but the contrast between 21” and 30” images did not reach the adjusted significance level [all *t*s ≤ 3.492; *p*s ≥ 0.002]. In addition, the increase from 10” to 15” scaled landscape images was not significant [*t* = −1.477; *p* = 0.153]. For details see Table S5 in the online supplementary file. In summary, we found a general increase of the number of fixations with increasing image size, supporting Hypothesis 3.

**Table 6:** Results of the 2 × 3 × 5 (image condition × image category × image size) ANOVA for the number of fixations.

Effect	*F*	*p*	*η*_*p*_^2^
**Main effects**			
Image condition	13.214	<0.005	0.365
Image category	136.844	<0.001	0.856
Image size	248.128	<0.001	0.915
**Two-way interactions**			
Image condition × image category	17.285	<0.001	0.429
Image condition × image size	3.371	<0.050	0.128
Image category × image size	15.740	<0.001	0.406
**Three-way interaction**			
Image condition × image category × image size	4.687	<0.001	0.169

Next, we determined the form of the increase of fixations by searching for an adequate estimate of the slope. Analogous to the analysis of saccade amplitudes, we used a lack-of-fit *F*-test to compare the curves of the observed number of fixations with a linear regression fitted to these data. This was done individually for each image category, and the results for the scaled and cropped conditions were analyzed separately.

In the scaled condition, the results showed no significant difference between the linear regression and the real data for urban and landscape images [both *F*s ≤ 1.821, *p*s ≥ 0.147, *R* ^2^ ≥ 0.244]. This suggests that the increase of the number of fixations for urban and landscape images could be explained in terms of a linear trend. However, as shown in Fig. 7a, the curve for landscape images showed a slight concavity. Although our linear regression for landscape images already provided a good model for describing the data, we computed the natural logarithm for each stimulus size and applied a linear regression to the log-transformed data. For landscape images, the results of the log-transformed model revealed no significant deviation from linearity [*F* = 0.378, *p* = 0.769, *R* ^2^ = 0.271] and provided an even better model than the linear model, as indicated by a slightly higher correlation [linear model: *R* ^2^ = 0.244; log-linear model: *R* ^2^ = 0.271]. For webpages, the lack-of-fit *F*-test revealed a significant deviation from linearity with regard to the original linear model [*F* = 6.804, *p* ≤ 0.001, *R* ^2^ = 0.575]. Consequently, the increase of the number of fixations observed for webpages could not be explained by a linear model. In fact, Fig. 7a shows that the curve for webpages also tends to follow a logarithmic rather than a linear trend. Therefore, we again applied a linear regression to the log-transformed data for webpages and found no significant difference between the models [*F* = 0.926, *p* = 0.431, *R* ^*2*^ = 0.630]. Hence, the log-transformed linear regression model well described the increase of the number of fixations with increasing image size.

In the cropped condition, the lack-of-fit *F*-test revealed a significant deviation from linearity for webpages [*F* = 4.802, *p* = 0.003, *R* ^*2*^ = 0.478] and urban images [*F* = 2.821, *p* = 0.042, *R* ^*2*^ = 0.418], but not for landscape images [*F* = 1.561, *p* = 0.203, *R* ^*2*^ = 0.245]. Thus, the increase of the number of fixations for webpages and urban images did not follow a linear trend. Therefore, we applied a linear regression to the log-transformed data for each category. The results revealed no significant deviation from linearity for webpages or urban images [both *F*s ≤ 0.767, *p*s ≤ 0.515, *R* ^2^ ≥ = 0.451]. Further, the deviation from linearity in the log-transformed data also turned out not to be significant for landscape images [*F* = 0.772, *p* = 0.512, *R* ^2^ = 0.260]. Correlation scores indicated that the log-transformed regression described the increase of the number of fixations for landscape images better than the original linear regression [linear model: *R* ^2^ = 0.245; log-linear model: *R* ^2^ = 0.260]. Thus, the number of fixations increased logarithmically with increasing image size in all image categories in the cropped condition.

However, a higher number of fixations on larger images is not necessarily linked to higher visual exploration within the image. For example, a magnification of details (scaled condition) in large images could lead to a re-fixation of already seen image regions. This leads to a higher number of fixations without an increase of image exploration (see schematic example in Fig. 7b). Note, that this approach differs from the previous entropy analysis, in which we showed that visual exploration increased in terms of a higher expansion of fixations. The expansion in entropy was a result of a larger spatial dimension of large images and thus larger distances between fixated regions of interest. In the current approach, we measured the exploration within the image in terms of the number of fixations (Fig. 7b). To answer whether the increase of fixations on large images is linked to a spatially more extensive exploration of the image (i.e. more fixated regions of interest), we calculated the number of image regions that have been fixated on each image size and category for scaled and cropped condition. Following previous studies^23, 63, 64^ we applied a 5 × 5 grid to each image, resulting in 25 equally sized rectangular image regions. We then calculated the number of fixated regions of each image and finally averaged over all images of the same size and category. As the size of the 25 image regions scaled with varying image size, a higher number of fixated image regions thus shows a larger spatial exploration of the images.

We computed a 2 × 3 × 5 (image condition × image category × image size) ANOVA with the number of fixated regions as the dependent variable. We suggested, that more image regions had to be fixated on larger images in order to show an increase of image exploration with increasing image size. The results of the ANOVA revealed the predicted main effect of image size. However, we also found that this main effect was qualified by image category and image condition, indicated by significant two-way and three-way interactions (Table 7). As shown in Fig. 7c, we did not find a continuous increase of the number of fixated regions with increasing image size. Using paired *t*-tests (Bonferroni-adjusted alpha level: *α* = 0.0008), we found a significant increase of the number of fixated image regions in the scaled condition from 7” to 30” landscape images and webpages [both *t*s ≥ 4.896; *p*s < 0.0008]; some more contrasts reached statistical significance (see Table S6 in the online supplementary file). On scaled urban images, the number of fixated image regions did not significantly change with increasing image size [all *t*s ≤ 2.745; *p*s ≥ 0.012]. In the cropped condition, we found no significant difference of the fixated image regions across image sizes in all categories [all *t*s ≤ 3.651; *p*s ≥ 0.001], except an increase of fixated image regions from 7” to 21” cropped urban and landscape images [both *t*s ≥ 4.311; *p*s < 0.0008].

**Table 7:** Results of the 2 × 3 × 5 (image condition × image category × image size) ANOVA for a number of fixated image regions.

Effect	*F*	*p*	*η*_*p*_^2^
**Main effects**			
Image condition	59.223	<0.001	0.720
Image category	10.832	<0.005	0.320
Image size	17.429	<0.001	0.431
**Two-way interactions**			
Image condition × image category	22.169	<0.001	0.491
Image condition × image size	8.557	<0.001	0.271
Image category × image size	4.422	<0.005	0.161
**Three-way interaction**			
Image condition × image category × image size	3.156	<0.010	0.121

Overall, the number of fixated image regions increased with increasing image size on scaled webpages and landscape images, reflecting a more spatially extensive exploration within larger images. We did not find this increase on scaled urban images. In the cropped condition, the number of fixated image regions did not change across image sizes, with two exceptions. However, by looking at the definition of the cropped condition, we constantly added information to the periphery when increasing the image size. In contrast, the sizes of the image regions we used in our analysis scaled linearly with the image size to maintain the same amount of image regions (*n* = 25). Consequently, on larger images in the cropped condition, the individual image regions contained more information of the scenery. Therefore, the constant number of fixated image regions across varying image sizes that we observed indicates more exploration in the cropped condition.

In conclusion, the initially observed increase in the number of fixations on larger images coincided with a more spatially extensive exploration in both image conditions and all image categories, except scaled urban images.

**Figure 8:**
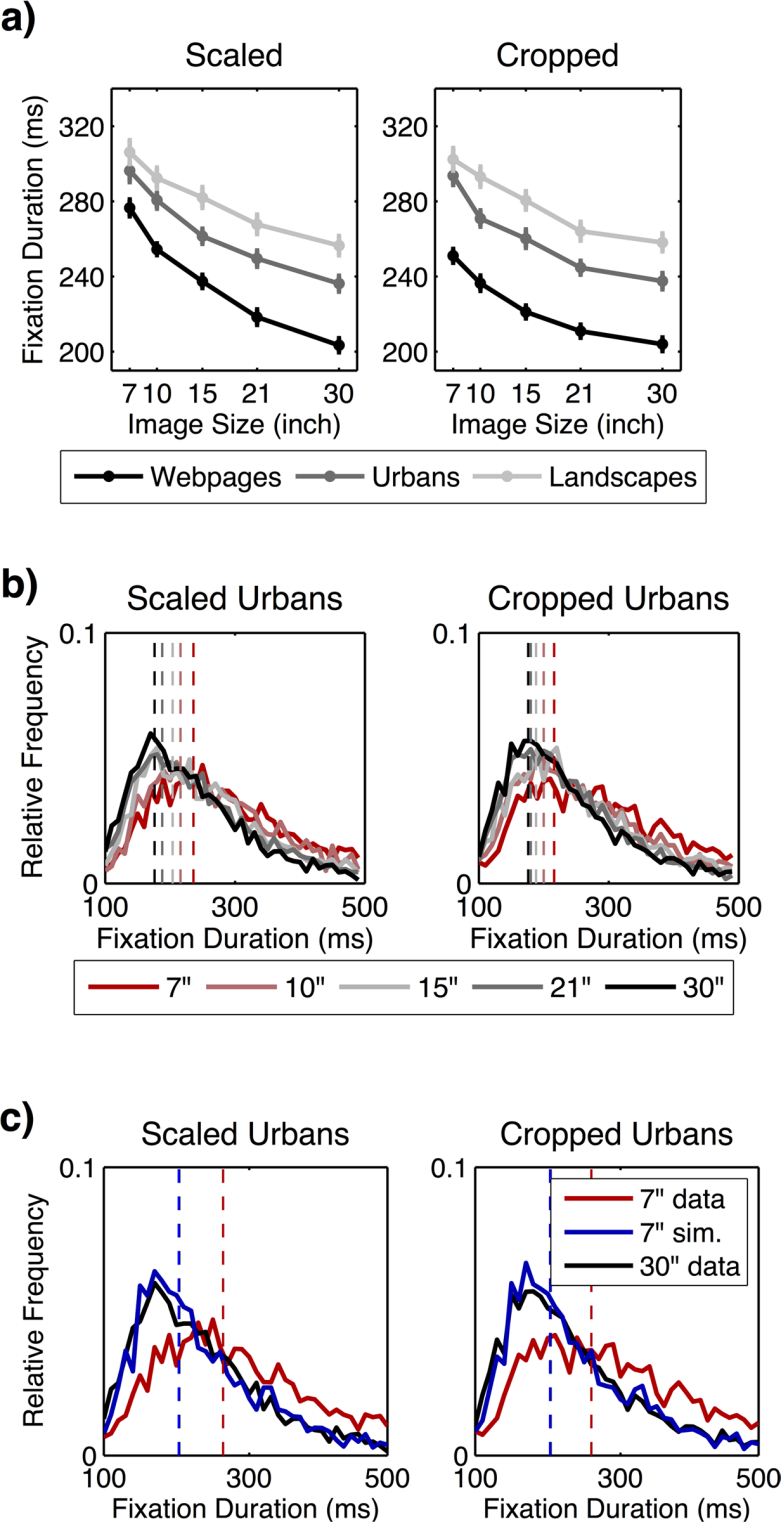
(**a**) Decrease of the mean fixation duration across image sizes for all image categories depending on scaled and cropped conditions. Error bars indicate the standard error of the mean. (**b**) Real distribution of fixation durations for urban images depending on image size and condition (scaled vs. cropped). (**c**) Real distribution of fixation durations on 7” and 30” images as well as the simulated distribution for 7” urban images based on fixations sampled from 30” urban images.

Hypothesis 3 also stated that the exploitation decreases with increasing image size, which is indicated by shorter fixation durations. Accordingly, we examined the mean duration of individual fixations depending on image category and image size. We computed a 2 × 3 × 5 (image condition × image category × image size) ANOVA with the mean fixation duration as the dependent variable. As predicted, we found a main effect of the image size additionally qualified by image category and image condition (see Table 8 and Fig. 8a). Paired *t*-tests (Bonferroni-adjusted alpha level: *α* = 0.0008) revealed a continuous decrease in fixation durations on scaled webpages and urban images [all *t*s ≥ 4.504; *p*s < 0.0008]. On scaled landscape images as well as on cropped images of all categories we also found a continuous decrease in fixation durations with increasing image size, but some contrasts did not reach the adjusted significance level (see Table S7 in online supplementary file). Overall, fixation durations decreased constantly with stimulus size in both image conditions and all image categories.

**Table 8:** Results of the 2 × 3 × 5 (image condition × image category × image size) ANOVA for fixation duration.

Effect	*F*	*p*	*η*_*p*_^2^
**Main effects**			
Image condition	57.653	<0.001	0.715
Image category	206.799	<0.001	0.900
Image size	243.015	<0.001	0.914
**Two-way interactions**			
Image condition × image category	24.018	<0.001	0.511
Image condition × image size	7.292	<0.005	0.241
Image category × image size	3.904	<0.005	0.145
**Three-way interactions**			
Image condition × image category × image size	3.876	<0.005	0.144

Next, we investigated the form of the decrease in fixation durations. We again started with a linear regression for the data. In the scaled condition, the lack-of-fit *F*-test revealed a significant deviation from linearity for webpages [*F* = 4.528, *p* = 0.005, *R* ^2^ = 0.616], but not for urban and landscape images [both *F*s ≤ 2.219, *p*s ≥ 0.090, *R* ^2^ ≥ 0.279]. This result showed that the linear model was already sufficient for describing the decrease in fixation durations for urban and landscape images. However, as shown in Fig. 8a, we observed a flattening of the curves with increasing image size for all image categories. Hence, we calculated the natural logarithm for each image size and applied a linear regression to the log-transformed data. Results of the lack-of-fit *F*-test were not significant for any of the image categories [all *F*s ≤ 0.298, *p*s ≥ 0.827, *R* ^2^ ≥ 0.290], indicating that the log-transformed model also adequately described the data. Moreover, it turned out to be a slightly more precise model for urban and landscape images than the original linear regression model, as indicated by higher correlations [urban linear model: *R* ^2^ = 0.433; urban log-linear model: *R* ^2^ = 0.463; landscape linear model: *R* ^2^ = 0.279; landscape log-linear model: *R* ^2^ = 0.290]. Hence, the mean fixation duration decreased rather logarithmically with increasing stimulus size for all image categories in the scaled condition.

In the cropped condition, the results of the lack-of-fit *F*-test revealed a significant deviation from linearity for webpages [*F* = 3.800, *p* = 0.012, *R* ^2^ = 0.425] and urban images [*F* = 4.034, *p* = 0.009, *R* ^2^ = 0.395], but not for landscape images [*F* = 1.066, *p* = 0.366, *R* ^2^ = 0.281]. This indicates that the decrease in the mean duration of fixations on webpages and urban images did not follow a linear trend, as shown in Fig. 8a. Again, we computed the natural logarithm for each image size and applied a linear regression to the log-transformed data. We found no significant deviation from linearity for any of the image categories [all *F*s ≤ 1.018, *p*s ≥ 0.387, *R* ^2^ ≥ 0.295]. Moreover, the log-linear model turned out to describe the decrease in fixation durations for landscape images slightly better than the original linear model [linear model: *R* ^2^ = 0.281; log-linear model: *R* ^2^ = 0.295].

Overall, the mean duration of fixations decreased with increasing stimulus size in all image categories in both the scaled and the cropped condition, whereby the decrease was best described by a logarithmic trend.

In addition to the above observations, fixation durations might be influenced by other parameters. First, previous studies showed mixed results about the dependency between the duration of a fixation and its pre-saccadic amplitude^16, 52^. Second, as shown in our analysis above, larger images led to longer saccade amplitudes. Also, an increase of saccade amplitudes leads to longer durations of saccades^53, 54^. Hence, in this study, larger image sizes might have led to a larger fraction of time spent on saccades. In summation, this higher duration of saccades could reduce the remaining time available for fixations, as the temporal stimulus presentation was fixed across all image sizes. This reduction of time could be the reason why the increase in the number of fixations saturated on large images (cf. Fig. 7a). This in turn would then explain the logarithmic decrease that we found regarding fixation durations. To get a deeper understanding of the fixation duration results, we thus examined this issue in more detail.

First, we focused on the dependency between fixation durations and pre-saccadic amplitudes, by analyzing the distribution of fixation durations for all images. Based on the measured data, we calculated the frequencies of the mean fixation duration for each image category and size (Fig. 8b). The frequencies were divided into 40 bins covering the range from 100ms to 500ms. We normalized these frequencies with regard to the total number of fixations. Then, we applied the same simulation model as previously used for the distribution of saccade amplitudes (see above). Specifically, we applied randomly selected saccades from 30” images to randomly selected fixations from 7” images. For each valid (i.e., non-rejected) saccade we extracted the duration of the pre-saccadic fixation and calculated the corresponding frequency distribution. Importantly, given that this simulation process adequately predicted the distribution of small saccade amplitudes on the basis of the amplitudes for large images (see results above), the same will hold for fixation durations if they are tied to saccade amplitudes. Using chi-square tests and a model that was closely analogous to the variable truncation model for saccade amplitudes, we analyzed the real distribution of fixation durations found for 7” images and 30” images as well as the simulated distribution for 7” images based on data from the 30” images. This analysis was done separately for each image category, and the data was further separated for the scaled and cropped condition.

In the scaled condition, as exemplarily shown for urban images in Fig. 8c, comparing the simulated distribution of fixation durations with the real distribution for 7” images revealed a strong and significant effect for each image category [all χ^2^ > 124.518, *ps < *0.001], indicating a significant difference between modeled and actually observed data. The comparison of the simulated distribution and the distribution of fixation durations found for 30” images did not show a significant difference in any of the image categories [all χ^2^ < 32.852, *p*s ≥ 0.745]. Therefore, the simulated distribution of fixation durations for 7” images, extracted from 30” images, could not predict the real data found for 7” images; instead, it matched the distribution found for 30” images.

In the cropped condition, the simulated distribution for 7” images also strongly and significantly differed from the distribution actually found for 7” images of all categories [all χ^2^ ≥ 103.459, *p*s < 0.001]. By contrast, when comparing the simulated distribution for 7” images with the distribution observed for 30” images, no significant difference occurred for any of the image categories [all χ^2^ ≤ 28.539, *p*s ≥ 0.891]. Thus, as for scaled images, the simulated distribution of fixation durations for 7” images could not predict the real data found for 7” images; instead, it matched the distribution found for 30” images.

Overall, the simulation model based on saccade amplitudes did not explain the change in fixation durations across image sizes (cf. Figs 5d and 8c). The decrease in fixation durations with increasing image size was not a result of a dependency between saccade amplitude and fixation duration. Saccade amplitude and fixation duration were independent of each other.

**Figure 9:**
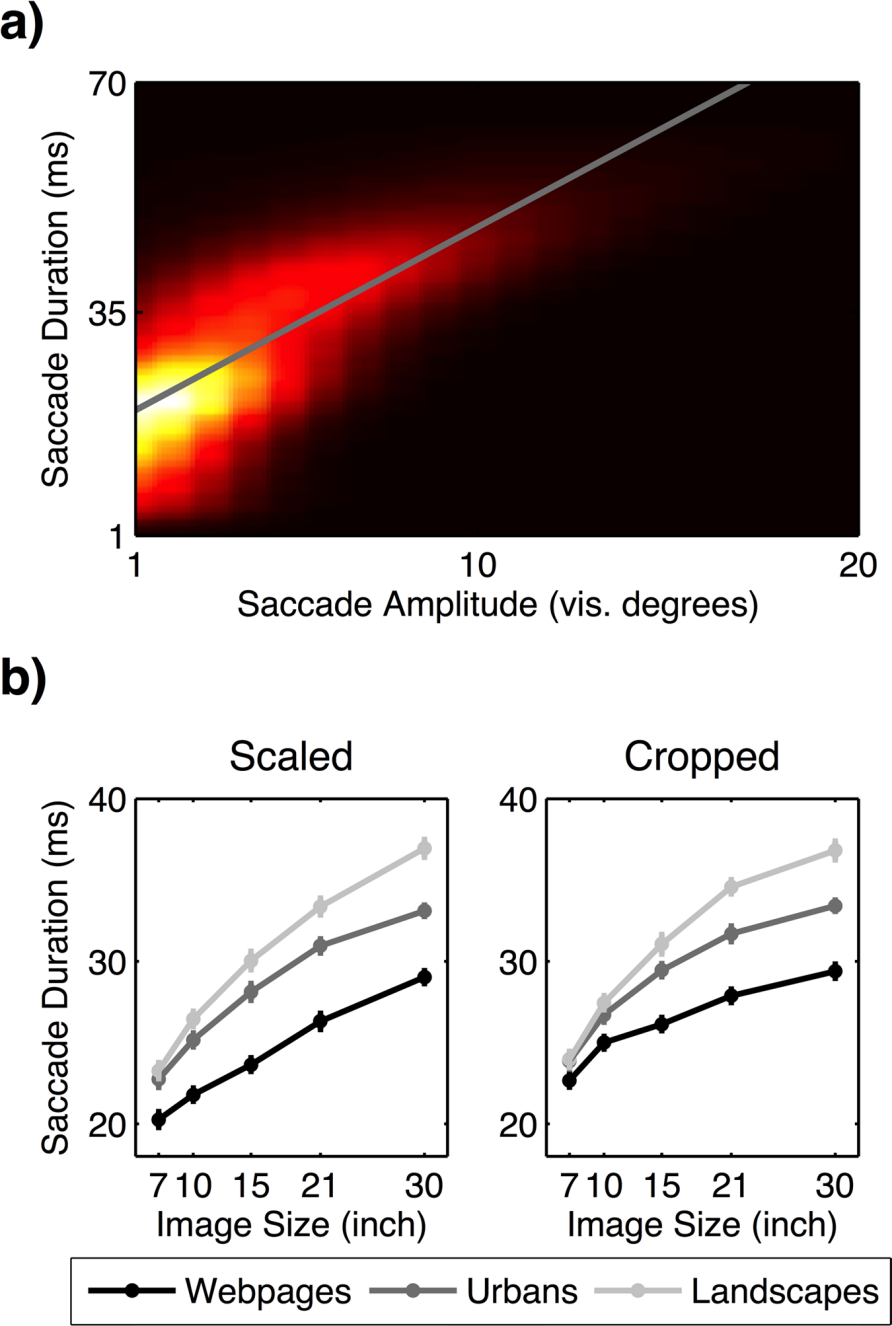
(**a**) Positive correlation between the amplitude and duration of a saccade. (**b**) The increase of the mean saccade duration across image sizes depending on image category and condition (scaled vs. cropped). Error bars indicate standard error of the mean.

In the next step, we focused on the influence of saccade durations on the logarithmic decrease of fixation durations. We initially computed the saccade amplitude and its respective duration for each saccade in our experiment. To exclude outliers, we used the same procedure as for fixation durations. We excluded saccade durations that were two standard deviations above the grand mean (cut-off: 64.38ms; 3.02% of the data). Further, we excluded saccades that targeted or originated outside of the respective image size (3.37% of the data). Using a Pearson correlation test, we found a positive linear dependency between saccade amplitudes and saccade durations [*R* ^*2*^ = 0.820, *p* < 0.001]. Longer amplitudes led to longer durations of saccades (Fig. 9a).

Next, we calculated the average saccade duration for each image category and image size and computed a 2 × 3 × 5 (image condition × image category × image size) ANOVA. We suggested that larger images led to longer saccade durations due to longer saccade amplitudes. As predicted, the ANOVA revealed a main effect of the image size that was additionally qualified by image category and image condition (Fig. 9b and Table 9). Paired *t*-tests (Bonferroni-adjusted alpha level: *α* = 0.0008) showed that saccade durations continuously increased with increasing image size in all image categories and both image conditions [all *t*s ≥ 4.265; *p*s < 0.0008]. The significant interactions reflected different effect sizes. Results for all contrasts are depicted in Table S8 in the online supplementary file. Overall, we found that saccade durations increased with increasing image size in all image categories of both image conditions.

**Table 9:** Results of the 2 × 3 × 5 (image condition × image category × image size) ANOVA for saccade durations.

Effect	*F*	*p*	*η*_*p*_^2^
**Main effects**			
Image condition	220.622	<0.001	0.906
Image category	221.057	<0.001	0.906
Image size	472.506	<0.001	0.954
**Two-way interactions**			
Image condition × image category	25.137	<0.001	0.522
Image condition × image size	16.927	<0.001	0.424
Image category × image size	38.448	<0.001	0.626
**Three-way interaction**			
Image condition × image category × image size	2.384	<0.050	0.094

Finally, with a lack of fit *F*-test, we could show that the increase of saccade durations with image size followed a logarithmic trend in each image category of both, the scaled and cropped condition [all *F*s ≤ 1.743, *p*s ≥ 0.162, *R* ^2^ ≥ 0.575].

Overall, we saw that larger images indeed led to longer saccade durations as an effect of longer saccade amplitudes. This effect followed a logarithmic trend in all image categories in the scaled and cropped. Therefore, the time available for doing fixations, given the fixed stimulus presentation duration, was reduced when observing large images. This effect influenced the duration of fixations. However, the difference in saccade durations across image sizes was in the order of magnitude of 20 ms, much lower compared to the average change in fixation durations (cf. Figs 8a and 9b). The overall time spent on saccades was on average 277.43 ms longer on 30” images compared to 7” images. This time describes a number of 1–2 fixations that had to be sacrificed on 30” images due to higher saccade durations. But even given this effect, the total number of fixations increased on larger images. Furthermore, such a small number was not sufficient to explain the observed decrease in fixation durations with larger images. Concluding, we assume that although saccade durations increased with image size, they have by themselves only a limited effect on the number and duration of fixations.

To summarize, the decrease of fixation duration with increasing image sizes did not depend on the saccade amplitudes and only were weakly affected by saccade durations and latencies. For the major part, the decrease of fixation durations was a consequence of an increasing number of fixations and thus increased visual exploration fully compatible with Hypothesis 3.

## Discussion

In the present study, we used stimuli with varying spatial properties and sizes to explore the trade-off between exploration and exploitation. Specifically, introducing larger stimuli increased the demands on sampling different regions by a larger number of widely distributed fixations (exploration). Given fixed time constraints, this establishes a limitation to the time available for focused attention to local regions (exploitation). Here we could characterize this trade-off with respect to commonly used eye-tracking parameters.

We found that the spatial bias in terms of central tendency and entropy was significantly influenced by image size: the central tendency scaled mainly linearly with the image size in both the scaled and cropped image conditions. This scaling was comparable for all image categories. The entropy, giving the spatial distribution of fixations independent of specific geometrical arrangements, increased in the scaled and cropped conditions for all image categories, but not in an exclusively logarithmic fashion. These results fully support Hypothesis 1 proposing that visual exploration increases with an increase of the image size. The mean saccadic amplitude scaled linearly with the image size in all categories in both the scaled and cropped image conditions. Only in cropped webpages did the increase in saccade amplitude follow a logarithmic trend. These results nominally support the Hypothesis 2 proposing that visual exploration is supported by an increase of saccadic amplitudes. However, the data can be well described by the variable truncation model, which operates on a constant basic distribution of saccadic amplitudes. Moreover, in all image categories, the number of fixations increased with stimulus size. Except for scaled urban pictures, this increase followed a logarithmic trend with increasing image size. We saw, that the increased number of fixations indeed led to a spatially more extensive exploration within larger images in all image categories of both conditions, except for scaled urban images. Visual exploitation, which was indicated by fixation duration, decreased with increasing image size in all conditions in a logarithmic trend. We found that saccadic amplitude and duration of single fixations were independent of each other and that saccade durations only weakly affected fixation durations. These results fully support Hypothesis 3 of reduced exploitation with increasing image size. All in all, visual exploration robustly scaled with the image size, indicating a shift from exploitation to exploration on larger images.

As a control, we performed all experiments with two complementary image manipulations. Smaller images were either generated by scaling down the original, or by cropping a region of desired size. The omnibus statistical analyses revealed that the effect of image size was qualified by image condition regarding all eye movement parameters. However, the main results were significant and similar in both the scaled and the cropped condition. This demonstrates that visual behavior was affected by image size (and image category) in a similar way, regardless of the varying depth and resolution of details within visual scenes. We therefore conclude that spatial properties of the image in terms of its size are a crucial factor to affect visual behavior.

The present results support our general hypothesis that, in terms of the spatial bias, visual exploration scales with varying image sizes. We found changes in central tendency as well entropy with increasing image size. Tatler^44^ argued that the central tendency in natural images might arise independently of image features within the scene. In his view, the tendency to look at the center of an image on the monitor stems from two sources: spatial viewing properties that situate the optimal viewing position at the center of the screen and constraints of the oculomotor system. As both aspects of spatial bias were similar for both scaled and cropped images, it is unlikely that properties of the distribution of local features drives this effect. Therefore, our results support the view that the spatial bias originates from the oculomotor system. However, we showed that the image size affects the central tendency in such a way that it adapted adequately with the increasing size of the presented image. In calculating the entropy, we found that the geometrically independent distribution of fixations also increased with increasing image size. Overall, we showed that the size of the presented stimulus seems to be a crucial spatial property that affects visual exploration in terms of the distribution of fixations on images.

In order to further investigate how image size affects visual behavior, we evaluated the amplitudes of saccades. Supporting a previous study^48^, we found an overall linear increase in saccade amplitude with increasing image size. However, humans in general tend to exhibit small saccade amplitudes for natural images^40, 44^. This leads to a distribution of saccade amplitudes that follows a log-normal trend^40–43^. This tendency toward small saccades has been suggested to originate from motor biases in the saccadic system^44^ and should be independent of inherent features of the visual scene. Indeed, we found that the distribution of saccade amplitudes showed a similar progression through each image size in every image category, with a high number of small saccades for small image sizes followed by a small number of larger saccades for large image sizes. By extracting information on the scaled spatial bias for different image sizes with our simulation model, we could identify that the distribution of saccades for large images was compatible to the distribution of saccades for small images. This suggests that the higher mean saccade amplitude for large images mentioned above resulted from an interaction between the basic distribution of saccade amplitudes and the eccentricities of the spatial bias that changed according to the image size. Thus, in terms of the saccadic distribution, motor constraints remain remarkably constant across different image sizes, while only the variable truncation depends on the image size.

For a better understanding of the time available for focused attention to specific regions of the image, we analyzed the number of fixations and fixation durations for each image size. In doing so, we found that the number of fixations increased in a logarithmic fashion with increasing image size. Based on the changes in entropy and the grid analysis (i.e., number of fixated image regions), we showed that the increase of fixations was linked to a spatially more extensive exploration within the image in all conditions13.We found that fixation duration decreased in a negative logarithmic trend opposite to the number of fixations. As fixation durations are linked with the in-depth information processing of a fixated region of interest^14–16^, this shows that larger images lead to a reduced in-depth processing of sensory information from specific locations. This demonstrates a shift from exploitation to exploration of the scenery.

Previous studies have argued that information processing needs a certain minimum amount of time in order to allow for interpretation of the fixated region^65^. This establishes a lower threshold for fixation durations and prevents the complete compensation of increased exploration in larger images by reduced exploitation. With the simulated variable truncation model, we could exclude the explanation that the decrease in exploitation was a result of a correlation between saccade amplitude and the duration of their pre-saccadic fixation, as described in previous studies^16, 41, 66^. Further, we showed that a general increase of saccade durations based on larger saccade amplitudes in large image sizes did only weakly affect fixation durations. Thus, visual exploitation decreased in larger images due to an increase in visual exploration. As a consequence of a fixed temporal limitation of sensory information processing, the decrease of fixation durations followed a logarithmic dependence. This logarithmic trend led to a saturation of the number of fixations on large images.

We need to consider that our results could have been influenced by other parameters that were not taken into account in this study. Previous literature reported a logarithmic increase of saccade latencies in saccade amplitudes of 0.5° or less^67^. As smaller images led to a higher number of small saccades, a larger summation of saccade latencies could have increased the fixation durations for these small images. Although we doubt that the higher number of small saccades (the difference between 7” and 30”: 8.59%) had a significantly high impact on fixation durations, this point might be addressed in a further study.

Additionally, gaze behavior as natural viewing behavior is usually linked with a combination of eye and head movements during visual scene perception^68, 69^. The eye tracker employed in the present study measured the gaze direction by evaluating eye as well as head positions. Therefore, all presented results relate to gaze movements. As we instructed participants to maintain a stable head position, eye movements dominated the visual behavior and head movements had only a residual influence. Thus, our results and the definition of gaze behavior relate to eye movements and we maintain that terminology.

Furthermore, we were to some extent limited when changing the size of stimuli. Here, we focused on image sizes ranging between 7” and 30”, which is only a limited fraction of the total visual field of a healthy human. Therefore, we need to be careful when extending our findings to visual behavior in real-world scenarios^68, 70^. However, most of the screens in everyday use provide display sizes that match the stimulus sizes covered by our study. Thus, our results can indeed be applied to normal viewing behavior for different media devices. Further, our results suggest clear trends (linear and logarithmic) in the progress of commonly investigated eye-tracking parameters. Hence, not only can we make conclusions with respect to the screen sizes we used, but we can also predict the results likely to occur when using screens of other sizes. This is important for scientists conducting eye-tracking experiments with varying monitor sizes.

Another issue concerning our study design might be that we presented each image size blockwise. Participants therefore could have predicted the size of the following stimulus within one block and thus developed a certain viewing strategy. However, previous studies showed that repeated stimulus presentation does not necessarily affect visual behavior^23^. In addition, the blockwise presentation might reflect real-life scenarios better than a randomization of image sizes. Humans usually tend to watch images or browse webpages on the same screen for some time (e.g., laptop) before switching to another device (e.g., smartphone). Also in typical eye tracking experiments, participants observe a large number of same-sized images on the monitor. Thus, a blockwise presentation of image sizes in our experiment and therefore a possible adaptation of viewing strategies do follow typical visual behavior.

With respect to the choice of our image categories, we have to consider the difference between webpages and natural images (urban and landscape images) in more detail. We saw, inter alia, that the mean saccade amplitudes and fixation durations were much smaller on average for webpages than for the natural image categories. These effects might have been a result of small (albeit present) text elements in webpages. Instead of free exploration, subjects could have read these text elements, which led to smaller saccades and shorter fixation durations. Although we found that all parameters according to webpages showed a similar trend as for urban and landscape images when manipulating the image size, further studies might focus in more depth on how the presence of text elements in varying image size affects saccade amplitudes and fixation durations.

In general, our study provides a valuable baseline for investigating the trade-off between exploration and exploitation for different image sizes and, thus, for different spatial properties. Still, further research has to be done in order to investigate visual behavior with regard to different spatial properties of stimuli, such as movement^71, 72^, the interplay between image size and complexity of the scenery, and dynamic changes in the image size during observation.

## Conclusion

Researchers have investigated several bottom-up and top-down factors that affect visual behavior in natural viewing contexts, but evaluations of exploration and exploitation with regard to varying spatial properties are lacking. With the exception of one study^48^, image size has been neglected in most of the research so far. Here, we investigated the impact of such varying image sizes on visual behavior. In doing so, we showed that image size is a crucial factor that affects overt visual attention in terms of exploration and exploitation. Visual researchers should be aware of this additional image feature when relating their own data to the results of studies being conducted in a laboratory with a different display setup. It is especially important that the effect of the stimulus size is taken into account very carefully when comparing commonly used eye-tracking parameters across studies. Also in media, knowledge about how image size affects gaze behavior might be of importance, for example when designing new webpages optimized for devices with varying display sizes. In a nutshell, we found that image size has to be considered as an important spatial property that shifts the balance between exploration and exploitation in overt attention.

## Electronic supplementary material

Supplementary material
