# Supplementary material for: Exploration and Exploitation in Natural Viewing Behavior
Source: Sci Rep. 2017 May 23;7:2311. doi: 10.1038/s41598-017-02526-1 (PMC5442137; doi:10.1038/s41598-017-02526-1)
Supplement: Supplementary file 1 — Supplementary material [file 41598_2017_2526_MOESM1_ESM.doc]

**Exploration and Exploitation in Natural Viewing Behavior**

**Ricardo Ramos Gameiro^1*^, Kai Kaspar^1,2^, Sabine U. König^1^, Sontje Nordholt^1^, Peter König^1,3^**

1 Institute of Cognitive Science, University of Osnabrück, Germany

2 Social and Media Psychology, Department of Psychology, University of Cologne, Germany

3 Department of Neurophysiology and Pathophysiology, University Medical Center Hamburg

Eppendorf, Germany

***Correspondence:** Ricardo Ramos Gameiro, Institute of Cognitive Science, University of Osnabrück, Osnabrück, Germany

Email: rramosga@uni-osnabrueck.de

**Supplementary Table S1:** Individual contrasts between pairs of image sizes regarding the central tendency (measured by the standard deviation of the fixation distribution) in the *horizontal direction*.

| **Contrast**  **(image size vs. size)** | **Scaled images** | |  | **Cropped images** | |
| --- | --- | --- | --- | --- | --- |
|  | ***t*** | ***p*** |  | ***t*** | ***p*** |
| **Webpages** |  |  |  |  |  |
| 7” vs. 10” | -14.150 | < .001* |  | -19.188 | < .001* |
| 7” vs. 15” | -18.315 | < .001* |  | -22.674 | < .001* |
| 7” vs. 21” | -31.864 | < .001* |  | -30.693 | < .001* |
| 7” vs. 30” | -23'157 | < .001* |  | -20.944 | < .001* |
| 10” vs. 15” | -12.394 | < .001* |  | -17.383 | < .001* |
| 10” vs. 21” | -25.667 | < .001* |  | -27.766 | < .001* |
| 10” vs. 30” | -22.711 | < .001* |  | -18.755 | < .001* |
| 15” vs. 21” | -14.267 | < .001* |  | -17.022 | < .001* |
| 15” vs. 30” | -20.178 | < .001* |  | -14.567 | < .001* |
| 21” vs. 30” | -12.805 | < .001* |  | -6.671 | < .001* |
| **Urban images** |  |  |  |  |  |
| 7” vs. 10” | -18.184 | < .001* |  | -14.522 | < .001* |
| 7” vs. 15” | -24.560 | < .001* |  | -21.074 | < .001* |
| 7” vs. 21” | -30.712 | < .001* |  | -31.771 | < .001* |
| 7” vs. 30” | -30.185 | < .001* |  | -24.968 | < .001* |
| 10” vs. 15” | -15.793 | < .001* |  | -16.605 | < .001* |
| 10” vs. 21” | -27.572 | < .001* |  | -29.274 | < .001* |
| 10” vs. 30” | -28.225 | < .001* |  | -24.743 | < .001* |
| 15” vs. 21” | -22.496 | < .001* |  | -20.309 | < .001* |
| 15” vs. 30” | -28.821 | < .001* |  | -22.098 | < .001* |
| 21” vs. 30” | -20.600 | < .001* |  | -15.761 | < .001* |
| **Landscape images** |  |  |  |  |  |
| 7” vs. 10” | -12.467 | < .001* |  | -13.257 | < .001* |
| 7” vs. 15” | -17.110 | < .001* |  | -17.679 | < .001* |
| 7” vs. 21” | -19.135 | < .001* |  | -21.488 | < .001* |
| 7” vs. 30” | -19.376 | < .001* |  | -19.923 | < .001* |
| 10” vs. 15” | -14.299 | < .001* |  | -13.840 | < .001* |
| 10” vs. 21” | -18.357 | < .001* |  | -21.029 | < .001* |
| 10” vs. 30” | -19.473 | < .001* |  | -19.630 | < .001* |
| 15” vs. 21” | -14.517 | < .001* |  | -17.618 | < .001* |
| 15” vs. 30” | -18.543 | < .001* |  | -18.266 | < .001* |
| 21” vs. 30” | -14.528 | < .001* |  | -14.173 | < .001* |

Note: * *p*-value is below the Bonferroni-adjusted alpha-level of .0008 applied due to multiple testing.

**Supplementary Table S2:** Individual contrasts between pairs of image sizes regarding central tendency (measured by the standard deviation of the fixation distribution) in the *vertical direction*.

| **Contrast**  **(image size vs. size)** | **Scaled images** | |  | **Cropped images** | |
| --- | --- | --- | --- | --- | --- |
|  | ***t*** | ***p*** |  | ***t*** | ***p*** |
| **Webpages** |  |  |  |  |  |
| 7” vs. 10” | -16.120 | < .001* |  | -16.975 | < .001* |
| 7” vs. 15” | -18.552 | < .001* |  | -29.618 | < .001* |
| 7” vs. 21” | -21.732 | < .001* |  | -25.985 | < .001* |
| 7” vs. 30” | -25.430 | < .001* |  | -22.369 | < .001* |
| 10” vs. 15” | -15.412 | < .001* |  | -15.432 | < .001* |
| 10” vs. 21” | -19.682 | < .001* |  | -21.402 | < .001* |
| 10” vs. 30” | -24.333 | < .001* |  | -20.414 | < .001* |
| 15” vs. 21” | -13.158 | < .001* |  | -11.195 | < .001* |
| 15” vs. 30” | -21.084 | < .001* |  | -15.147 | < .001* |
| 21” vs. 30” | -15.458 | < .001* |  | -11.185 | < .001* |
| **Urban images** |  |  |  |  |  |
| 7” vs. 10” | -11.306 | < .001* |  | -13.325 | < .001* |
| 7” vs. 15” | -19.773 | < .001* |  | -22.528 | < .001* |
| 7” vs. 21” | -25.730 | < .001* |  | -24.193 | < .001* |
| 7” vs. 30” | -20.875 | < .001* |  | -20.990 | < .001* |
| 10” vs. 15” | -12.355 | < .001* |  | -13.658 | < .001* |
| 10” vs. 21” | -24.400 | < .001* |  | -20.236 | < .001* |
| 10” vs. 30” | -19.960 | < .001* |  | -19.092 | < .001* |
| 15” vs. 21” | -12.876 | < .001* |  | -14.887 | < .001* |
| 15” vs. 30” | -17.052 | < .001* |  | -16.982 | < .001* |
| 21” vs. 30” | -11.995 | < .001* |  | -8.953 | < .001* |
| **Landscape images** |  |  |  |  |  |
| 7” vs. 10” | -10.763 | < .001* |  | -10.533 | < .001* |
| 7” vs. 15” | -14.954 | < .001* |  | -14.590 | < .001* |
| 7” vs. 21” | -19.190 | < .001* |  | -18.482 | < .001* |
| 7” vs. 30” | -20.958 | < .001* |  | -25.585 | < .001* |
| 10” vs. 15” | -11.456 | < .001* |  | -12.388 | < .001* |
| 10” vs. 21” | -17.338 | < .001* |  | -18.238 | < .001* |
| 10” vs. 30” | -20.596 | < .001* |  | -24.721 | < .001* |
| 15” vs. 21” | -14.205 | < .001* |  | -14.722 | < .001* |
| 15” vs. 30” | -18.845 | < .001* |  | -18.970 | < .001* |
| 21” vs. 30” | -13.854 | < .001* |  | -11.867 | < .001* |

Note: * *p*-value is below the Bonferroni-adjusted alpha-level of .0008 applied due to multiple testing.

**Supplementary Table S3:** Individual contrasts between pairs of image sizes regarding entropy.

| **Contrast**  **(image size vs. size)** | **Scaled images** | |  | **Cropped images** | |
| --- | --- | --- | --- | --- | --- |
|  | ***t*** | ***p*** |  | ***t*** | ***p*** |
| **Webpages** |  |  |  |  |  |
| 7” vs. 10” | -12.674 | < .001* |  | -12.506 | < .001* |
| 7” vs. 15” | -16.907 | < .001* |  | -14.793 | < .001* |
| 7” vs. 21” | -17.939 | < .001* |  | -14.269 | < .001* |
| 7” vs. 30” | -18.417 | < .001* |  | -14.755 | < .001* |
| 10” vs. 15” | -11.333 | < .001* |  | -11.578 | < .001* |
| 10” vs. 21” | -12.537 | < .001* |  | -10.987 | < .001* |
| 10” vs. 30” | -13.287 | < .001* |  | -12.063 | < .001* |
| 15” vs. 21” | -10.205 | < .001* |  | -7.688 | < .001* |
| 15” vs. 30” | -12.942 | < .001* |  | -10.081 | < .001* |
| 21” vs. 30” | -10.016 | < .001* |  | -8.826 | < .001* |
| **Urban images** |  |  |  |  |  |
| 7” vs. 10” | -13.898 | < .001* |  | -24.126 | < .001* |
| 7” vs. 15” | -25.361 | < .001* |  | -41.444 | < .001* |
| 7” vs. 21” | -28.585 | < .001* |  | -43.615 | < .001* |
| 7” vs. 30” | -29.202 | < .001* |  | -46.774 | < .001* |
| 10” vs. 15” | -17.223 | < .001* |  | -24.408 | < .001* |
| 10” vs. 21” | -25.654 | < .001* |  | -30.515 | < .001* |
| 10” vs. 30” | -27.693 | < .001* |  | -24.843 | < .001* |
| 15” vs. 21” | -13.443 | < .001* |  | -14.099 | < .001* |
| 15” vs. 30” | -18.678 | < .001* |  | -10.777 | < .001* |
| 21” vs. 30” | -9.550 | < .001* |  | -1.749 | .094 |
| **Landscape images** |  |  |  |  |  |
| 7” vs. 10” | -16.661 | < .001* |  | -17.477 | < .001* |
| 7” vs. 15” | -23.754 | < .001* |  | -23.663 | < .001* |
| 7” vs. 21” | -23.509 | < .001* |  | -29.317 | < .001* |
| 7” vs. 30” | -24.963 | < .001* |  | -30.241 | < .001* |
| 10” vs. 15” | -16.805 | < .001* |  | -13.063 | < .001* |
| 10” vs. 21” | -19.854 | < .001* |  | -21.064 | < .001* |
| 10” vs. 30” | -22.310 | < .001* |  | -23.156 | < .001* |
| 15” vs. 21” | -12.203 | < .001* |  | -10.606 | < .001* |
| 15” vs. 30” | -18.484 | < .001* |  | -12.279 | < .001* |
| 21” vs. 30” | -12.875 | < .001* |  | -8.094 | < .001* |

Note: * *p*-value is below the Bonferroni-adjusted alpha-level of .0008 applied due to multiple testing.

**Supplementary Table S4:** Individual contrasts between pairs of image sizes regarding saccade amplitudes.

| **Contrast**  **(image size vs. size)** | **Scaled images** | |  | **Cropped images** | |
| --- | --- | --- | --- | --- | --- |
|  | ***t*** | ***p*** |  | ***t*** | ***p*** |
| **Webpages** |  |  |  |  |  |
| 7” vs. 10” | -8.394 | < .001* |  | -13.929 | < .001* |
| 7” vs. 15” | -12.360 | < .001* |  | -15.892 | < .001* |
| 7” vs. 21” | -22.022 | < .001* |  | -25.218 | < .001* |
| 7” vs. 30” | -27.258 | < .001* |  | -22.203 | < .001* |
| 10” vs. 15” | -6.629 | < .001* |  | -7.228 | < .001* |
| 10” vs. 21” | -15.356 | < .001* |  | -16.794 | < .001* |
| 10” vs. 30” | -20.704 | < .001* |  | -17.284 | < .001* |
| 15” vs. 21” | -10.967 | < .001* |  | -10.601 | < .001* |
| 15” vs. 30” | -21.180 | < .001* |  | -17.004 | < .001* |
| 21” vs. 30” | -14.544 | < .001* |  | -7.085 | < .001* |
| **Urban images** |  |  |  |  |  |
| 7” vs. 10” | -14.875 | < .001* |  | -12.094 | < .001* |
| 7” vs. 15” | -19.731 | < .001* |  | -19.032 | < .001* |
| 7” vs. 21” | -31.478 | < .001* |  | -24.639 | < .001* |
| 7” vs. 30” | -29.233 | < .001* |  | -31.606 | < .001* |
| 10” vs. 15” | -10.454 | < .001* |  | -14.472 | < .001* |
| 10” vs. 21” | -23.060 | < .001* |  | -20.694 | < .001* |
| 10” vs. 30” | -25.424 | < .001* |  | -26.207 | < .001* |
| 15” vs. 21” | -14.120 | < .001* |  | -11.183 | < .001* |
| 15” vs. 30” | -23.839 | < .001* |  | -22.269 | < .001* |
| 21” vs. 30” | -14.513 | < .001* |  | -12.111 | .094 |
| **Landscape images** |  |  |  |  |  |
| 7” vs. 10” | -13.276 | < .001* |  | -14.910 | < .001* |
| 7” vs. 15” | -18.826 | < .001* |  | -16.767 | < .001* |
| 7” vs. 21” | -28.992 | < .001* |  | -30.137 | < .001* |
| 7” vs. 30” | -26.769 | < .001* |  | -27.872 | < .001* |
| 10” vs. 15” | -13.498 | < .001* |  | -10.524 | < .001* |
| 10” vs. 21” | -25.250 | < .001* |  | -23.270 | < .001* |
| 10” vs. 30” | -24.582 | < .001* |  | -24.266 | < .001* |
| 15” vs. 21” | -19.441 | < .001* |  | -19.740 | < .001* |
| 15” vs. 30” | -26.206 | < .001* |  | -25.583 | < .001* |
| 21” vs. 30” | -16.273 | < .001* |  | -14.475 | < .001* |

Note: * *p*-value is below the Bonferroni-adjusted alpha-level of .0008 applied due to multiple testing.

**Supplementary Table S5:** Individual contrasts between pairs of image sizes regarding the number of fixations.

| **Contrast**  **(image size vs. size)** | **Scaled images** | |  | **Cropped images** | |
| --- | --- | --- | --- | --- | --- |
|  | ***t*** | ***p*** |  | ***t*** | ***p*** |
| **Webpages** |  |  |  |  |  |
| 7” vs. 10” | -8.784 | < .001* |  | -5.586 | < .001* |
| 7” vs. 15” | -13.292 | < .001* |  | -12.733 | < .001* |
| 7” vs. 21” | -17.477 | < .001* |  | -14.041 | < .001* |
| 7” vs. 30” | -21.461 | < .001* |  | -15.764 | < .001* |
| 10” vs. 15” | -7.114 | < .001* |  | -6.434 | < .001* |
| 10” vs. 21” | -11.486 | < .001* |  | -9.858 | < .001* |
| 10” vs. 30” | -18.517 | < .001* |  | -11.168 | < .001* |
| 15” vs. 21” | -7.799 | < .001* |  | -4.791 | < .001* |
| 15” vs. 30” | -10.699 | < .001* |  | -6.515 | < .001* |
| 21” vs. 30” | -4.979 | < .001* |  | -2.773 | .011 |
| **Urban images** |  |  |  |  |  |
| 7” vs. 10” | -3.874 | < .001* |  | -4.302 | < .001* |
| 7” vs. 15” | -9.974 | < .001* |  | -8.521 | < .001* |
| 7” vs. 21” | -11.819 | < .001* |  | -13.281 | < .001* |
| 7” vs. 30” | -12.537 | < .001* |  | -11.297 | < .001* |
| 10” vs. 15” | -5.191 | < .001* |  | -5.812 | < .001* |
| 10” vs. 21” | -7.657 | < .001* |  | -14.096 | < .001* |
| 10” vs. 30” | -10.51 | < .001* |  | -12.041 | < .001* |
| 15” vs. 21” | -4.464 | < .001* |  | -4.847 | < .001* |
| 15” vs. 30” | -6.894 | < .001* |  | -6.181 | < .001* |
| 21” vs. 30” | -3.492 | .002 |  | -2.413 | .024 |
| **Landscape images** |  |  |  |  |  |
| 7” vs. 10” | -4.397 | < .001* |  | -6.018 | < .001* |
| 7” vs. 15” | -9.041 | < .001* |  | -6.743 | < .001* |
| 7” vs. 21” | -10.845 | < .001* |  | -10.580 | < .001* |
| 7” vs. 30” | -9.839 | < .001* |  | -9.007 | < .001* |
| 10” vs. 15” | -4.454 | < .001* |  | -1.477 | .153 |
| 10” vs. 21” | -6.738 | < .001* |  | -10.543 | < .001* |
| 10” vs. 30” | -6.643 | < .001* |  | -6.739 | < .001* |
| 15” vs. 21” | -4.121 | < .001* |  | -6.238 | < .001* |
| 15” vs. 30” | -5.565 | < .001* |  | -4.719 | < .001* |
| 21” vs. 30” | -1.140 | .024 |  | -0.719 | .479 |

Note: * *p*-value is below the Bonferroni-adjusted alpha-level of .0008 applied due to multiple testing.

**Supplementary Table S6:** Individual contrasts between pairs of image sizes regarding the number of fixated image regions.

| **Contrast**  **(image size vs. size)** | **Scaled images** | |  | **Cropped images** | |
| --- | --- | --- | --- | --- | --- |
|  | ***t*** | ***p*** |  | ***t*** | ***p*** |
| **Webpages** |  |  |  |  |  |
| 7” vs. 10” | -1.604 | .122 |  | -3.091 | .005 |
| 7” vs. 15” | -1.344 | .192 |  | -1.553 | .134 |
| 7” vs. 21” | -2.959 | .007 |  | -1.227 | .232 |
| 7” vs. 30” | -7.097 | < .001* |  | 0.831 | .415 |
| 10” vs. 15” | -0.210 | .835 |  | 1.213 | .238 |
| 10” vs. 21” | -1.526 | .141 |  | 0.915 | .369 |
| 10” vs. 30” | -5.538 | <.001* |  | 3.322 | .003 |
| 15” vs. 21” | -1.355 | .189 |  | 0.098 | .923 |
| 15” vs. 30” | -5.432 | <.001* |  | 2.870 | .009 |
| 21” vs. 30” | -3.858 | <.001* |  | 2.129 | .044 |
| **Urban images** |  |  |  |  |  |
| 7” vs. 10” | -0.595 | .557 |  | -2.919 | .008 |
| 7” vs. 15” | -1.911 | .068 |  | -3.463 | .002 |
| 7” vs. 21” | -2.597 | .016 |  | -4.311 | <.001* |
| 7” vs. 30” | -2.745 | .012 |  | -1.919 | .067 |
| 10” vs. 15” | -1.671 | .108 |  | -1.600 | .123 |
| 10” vs. 21” | -2.457 | .022 |  | -2.178 | .040 |
| 10” vs. 30” | -2.447 | .022 |  | -0.176 | .862 |
| 15” vs. 21” | -0.854 | .402 |  | -0.734 | .471 |
| 15” vs. 30” | -1.594 | .125 |  | 0.984 | .335 |
| 21” vs. 30” | -0.627 | .537 |  | 1.852 | .077 |
| **Landscape images** |  |  |  |  |  |
| 7” vs. 10” | -2.076 | .049 |  | -1.973 | .061 |
| 7” vs. 15” | -4.409 | <.001* |  | -2.067 | .050 |
| 7” vs. 21” | -4.775 | <.001* |  | -4.755 | <.001* |
| 7” vs. 30” | -4.896 | <.001* |  | -3.651 | .001 |
| 10” vs. 15” | -3.098 | .005 |  | 0.350 | .729 |
| 10” vs. 21” | -4.139 | <.001* |  | -3.196 | .004 |
| 10” vs. 30” | -4.208 | <.001* |  | -2.314 | .030 |
| 15” vs. 21” | -2.102 | .047 |  | -3.575 | .002 |
| 15” vs. 30” | -2.783 | .011 |  | -2.605 | .016 |
| 21” vs. 30” | -0.845 | .024 |  | -0.169 | .867 |

Note: * *p*-value is below the Bonferroni-adjusted alpha-level of .0008 applied due to multiple testing.

**Supplementary Table S7:** Individual contrasts between pairs of image sizes regarding fixation durations.

| **Contrast**  **(image size vs. size)** | **Scaled images** | |  | **Cropped images** | |
| --- | --- | --- | --- | --- | --- |
|  | ***t*** | ***p*** |  | ***t*** | ***p*** |
| **Webpages** |  |  |  |  |  |
| 7” vs. 10” | 5.934 | <.001* |  | 5.028 | <.001* |
| 7” vs. 15” | 12.892 | <.001* |  | 12.399 | <.001* |
| 7” vs. 21” | 18.577 | <.001* |  | 12.095 | <.001* |
| 7” vs. 30” | 17.882 | <.001* |  | 15.011 | <.001* |
| 10” vs. 15” | 11.284 | <.001* |  | 5.893 | <.001* |
| 10” vs. 21” | 12.070 | <.001* |  | 9.429 | <.001* |
| 10” vs. 30” | 19.301 | <.001* |  | 12.181 | <.001* |
| 15” vs. 21” | 7.043 | <.001* |  | 4.350 | <.001* |
| 15” vs. 30” | 12.872 | <.001* |  | 7.174 | <.001* |
| 21” vs. 30” | 6.963 | <.001* |  | 3.538 | .002 |
| **Urban images** |  |  |  |  |  |
| 7” vs. 10” | 4.545 | <.001* |  | 7.576 | <.001* |
| 7” vs. 15” | 8.489 | <.001* |  | 10.347 | <.001* |
| 7” vs. 21” | 10.394 | <.001* |  | 11.557 | <.001* |
| 7” vs. 30” | 9.831 | <.001* |  | 14.098 | <.001* |
| 10” vs. 15” | 6.008 | <.001* |  | 3.599 | .002 |
| 10” vs. 21” | 8.784 | <.001* |  | 8.514 | <.001* |
| 10” vs. 30” | 9.915 | <.001* |  | 11.223 | <.001* |
| 15” vs. 21” | 5.901 | <.001* |  | 4.698 | <.001* |
| 15” vs. 30” | 8.571 | <.001* |  | 7.114 | <.001* |
| 21” vs. 30” | 4.504 | <.001* |  | 3.223 | .004 |
| **Landscape images** |  |  |  |  |  |
| 7” vs. 10” | 4.389 | <.001* |  | 2.912 | .008 |
| 7” vs. 15” | 6.787 | <.001* |  | 8.522 | <.001* |
| 7” vs. 21” | 10.384 | <.001* |  | 10.344 | <.001* |
| 7” vs. 30” | 10.403 | <.001* |  | 9.947 | <.001* |
| 10” vs. 15” | 3.008 | .006 |  | 4.475 | <.001* |
| 10” vs. 21” | 8.173 | <.001* |  | 9.974 | <.001* |
| 10” vs. 30” | 8.085 | <.001* |  | 9.451 | <.001* |
| 15” vs. 21” | 7.009 | <.001* |  | 5.481 | <.001* |
| 15” vs. 30” | 7.627 | <.001* |  | 5.670 | <.001* |
| 21” vs. 30” | 3.177 | .004 |  | 1.693 | .104 |

Note: * *p*-value is below the Bonferroni-adjusted alpha-level of .0008 applied due to multiple testing.

**Supplementary Table S8:** Individual contrasts between pairs of image sizes regarding saccade durations.

| **Contrast**  **(image size vs. size)** | **Scaled images** | |  | **Cropped images** | |
| --- | --- | --- | --- | --- | --- |
|  | ***t*** | ***p*** |  | ***t*** | ***p*** |
| **Webpages** |  |  |  |  |  |
| 7” vs. 10” | -5.271 | < .001* |  | -10.088 | <.001* |
| 7” vs. 15” | -8.519 | <.001* |  | -11.297 | <.001* |
| 7” vs. 21” | -15.297 | <.001* |  | -16.255 | <.001* |
| 7” vs. 30” | -18.836 | <.001* |  | -21.150 | <.001* |
| 10” vs. 15” | -5.751 | <.001* |  | -4.265 | <.001* |
| 10” vs. 21” | -12.248 | <.001* |  | -10.232 | <.001* |
| 10” vs. 30” | -16.356 | <.001* |  | -14.977 | <.001* |
| 15” vs. 21” | -8.882 | <.001* |  | -7.728 | <.001* |
| 15” vs. 30” | -16.216 | <.001* |  | -14.103 | <.001* |
| 21” vs. 30” | -9.856 | <.001* |  | -5.562 | <.001* |
| **Urban images** |  |  |  |  |  |
| 7” vs. 10” | -8.925 | <.001* |  | -12.523 | <.001* |
| 7” vs. 15” | -14.286 | <.001* |  | -15.536 | <.001* |
| 7” vs. 21” | -22.786 | <.001* |  | -19.261 | <.001* |
| 7” vs. 30” | -19.890 | <.001* |  | -20.500 | <.001* |
| 10” vs. 15” | -7.589 | <.001* |  | -7.804 | <.001* |
| 10” vs. 21” | -16.655 | <.001* |  | -12.952 | <.001* |
| 10” vs. 30” | -17.467 | <.001* |  | -14.107 | <.001* |
| 15” vs. 21” | -9.083 | <.001* |  | -6.535 | <.001* |
| 15” vs. 30” | -12.992 | <.001* |  | -11.076 | <.001* |
| 21” vs. 30” | -5.931 | <.001* |  | -4.551 | <.001* |
| **Landscape images** |  |  |  |  |  |
| 7” vs. 10” | -8.748 | <.001* |  | -13.687 | <.001* |
| 7” vs. 15” | -12.967 | <.001* |  | -14.532 | <.001* |
| 7” vs. 21” | -17.866 | <.001* |  | -21.428 | <.001* |
| 7” vs. 30” | -21.642 | <.001* |  | -17.725 | <.001* |
| 10” vs. 15” | -11.288 | <.001* |  | -8.723 | <.001* |
| 10” vs. 21” | -16.692 | <.001* |  | -16.252 | <.001* |
| 10” vs. 30” | -21.505 | <.001* |  | -15.451 | <.001* |
| 15” vs. 21” | -8.462 | <.001* |  | -10.423 | <.001* |
| 15” vs. 30” | -16.763 | <.001* |  | -10.667 | <.001* |
| 21” vs. 30” | -9.835 | <.001* |  | -4.935 | <.001* |

Note: * *p*-value is below the Bonferroni-adjusted alpha-level of .0008 applied due to multiple testing.
